# Association of Intestinal Alkaline Phosphatase With Necrotizing Enterocolitis Among Premature Infants

**DOI:** 10.1001/jamanetworkopen.2019.14996

**Published:** 2019-11-08

**Authors:** Maya Heath, Rebecca Buckley, Zeromeh Gerber, Porcha Davis, Laura Linneman, Qingqing Gong, Brian Barkemeyer, Zhide Fang, Misty Good, Duna Penn, Sunyoung Kim

**Affiliations:** 1 Department of Pediatrics and Neonatology, Louisiana State University School of Medicine, Children’s Hospital of New Orleans, New Orleans; 2 Department of Biochemistry and Molecular Biology, Louisiana State University School of Medicine and Health Sciences Center, New Orleans; 3 Division of Newborn Medicine, Department of Pediatrics, Washington University School of Medicine in St Louis, St Louis Children’s Hospital, St Louis, Missouri; 4 Department of Biostatistics, Louisiana State University School of Public Health, New Orleans

## Abstract

**Importance:**

Necrotizing enterocolitis (NEC) in preterm infants is an often-fatal gastrointestinal tract emergency. A robust NEC biomarker that is not confounded by sepsis could improve bedside management, lead to lower morbidity and mortality, and permit patient selection in randomized clinical trials of possible therapeutic approaches.

**Objective:**

To evaluate whether aberrant intestinal alkaline phosphatase (IAP) biochemistry in infant stool is a molecular biomarker for NEC and not associated with sepsis.

**Design, Setting, and Participants:**

This multicenter diagnostic study enrolled 136 premature infants (gestational age, <37 weeks) in 2 hospitals in Louisiana and 1 hospital in Missouri. Data were collected and analyzed from May 2015 to November 2018.

**Exposures:**

Infant stool samples were collected between 24 and 40 or more weeks postconceptual age. Enrolled infants underwent abdominal radiography at physician and hospital site discretion.

**Main Outcomes and Measures:**

Enzyme activity and relative abundance of IAP were measured using fluorometric detection and immunoassays, respectively. After measurements were performed, biochemical data were evaluated against clinical entries from infants’ hospital stay.

**Results:**

Of 136 infants, 68 (50.0%) were male infants, median (interquartile range [IQR]) birth weight was 1050 (790-1350) g, and median (IQR) gestational age was 28.4 (26.0-30.9) weeks*.* A total of 25 infants (18.4%) were diagnosed with severe NEC, 19 (14.0%) were suspected of having NEC, and 92 (66.9%) did not have NEC; 26 patients (19.1%) were diagnosed with late-onset sepsis, and 14 (10.3%) had other non–gastrointestinal tract infections. For severe NEC, suspected NEC, and no NEC samples, median (IQR) fecal IAP content, relative to the amount of IAP in human small intestinal lysate, was 99.0% (51.0%-187.8%) (95% CI, 54.0%-163.0%), 123.0% (31.0%-224.0%) (95% CI, 31.0%-224.0%), and 4.8% (2.4%-9.8%) (95% CI, 3.4%-5.9%), respectively. For severe NEC, suspected NEC, and no NEC samples, median (IQR) enzyme activity was 183 (56-507) μmol/min/g (95% CI, 63-478 μmol/min/g) of stool protein, 355 (172-608) μmol/min/g (95% CI, 172-608 μmol/min/g) of stool protein, and 613 (210-1465) μmol/min/g (95% CI, 386-723 μmol/min/g) of stool protein, respectively. Mean (SE) area under the receiver operating characteristic curve values for IAP content measurements were 0.97 (0.02) (95% CI, 0.93-1.00; *P* < .001) at time of severe NEC, 0.97 (0.02) (95% CI, 0.93-1.00; *P* < .001) at time of suspected NEC, 0.52 (0.07) (95% CI, 0.38-0.66; *P* = .75) at time of sepsis, and 0.58 (0.08) (95% CI, 0.42-0.75; *P* = .06) at time of other non–gastrointestinal tract infections. Mean (SE) area under the receiver operating characteristic curve values for IAP activity were 0.76 (0.06) (95% CI, 0.64-0.86; *P* < .001), 0.62 (0.07) (95% CI, 0.48-0.77; *P* = .13), 0.52 (0.07) (95% CI, 0.39-0.67; *P* = .68), and 0.57 (0.08) (95% CI, 0.39-0.69; *P* = .66), respectively.

**Conclusions and Relevance:**

In this diagnostic study, high amounts of IAP protein in stool and low IAP enzyme activity were associated with diagnosis of NEC and may serve as useful biomarkers for NEC. Our findings indicated that IAP biochemistry was uniquely able to distinguish NEC from sepsis.

## Key Points

**Question:**

Unlike candidate biomarkers inclusive for all forms of systemic inflammation, can dysfunction in host management of microbiota have a high positive predictive value as a biomarker for necrotizing enterocolitis?

**Findings:**

In this diagnostic study of 136 premature infants, high amounts of intestinal alkaline phosphatase protein in stool and low intestinal alkaline phosphatase enzyme activity were associated with diagnosis of necrotizing enterocolitis. There was no association of intestinal alkaline phosphatase measures with non–gastrointestinal tract infections.

**Meaning:**

Measuring the inability of intestinal alkaline phosphatase to maintain host-microbiota homeostasis can potentially guide decisions for personalized care and treatment when an infant is most susceptible to developing necrotizing enterocolitis.

## Teaser

This diagnostic study evaluates whether aberrant intestinal alkaline phosphatase biochemistry in infant stool is a molecular biomarker for necrotizing enterocolitis and not associated with sepsis in the absence of necrotizing enterocolitis.

## Introduction

Necrotizing enterocolitis (NEC) is a common neonatal gastrointestinal (GI) tract emergency with a high mortality rate^1^ and long-term morbidities, including short-gut syndrome, nutritional deficiency, and neurodevelopmental delay.^2,3^ Suspected NEC presents with mild, nonspecific symptoms that frequently resolve with minimal intervention; no clinical test is an established criterion standard for suspected NEC. Radiographic evidence, such as pneumatosis intestinalis, is used to diagnose severe or advanced disease but has a sensitivity as low as 44%,^4^ has limited specificity,^5^ and lacks concordance in interpretation.^6,7,8^

**Figure 1.**
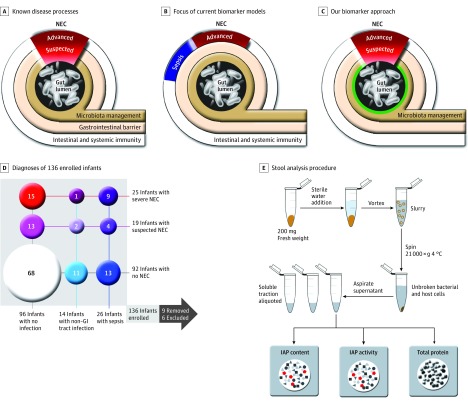
Association of Necrotizing Enterocolitis (NEC) and Late-Onset Sepsis With Gut Defense Mechanisms A-C, Physiological and structural changes in the gut, associated with NEC, are overlaid in the cross-sectional view of the small intestine. Research efforts to develop an NEC biomarker has focused on proteins in immunity cascades and in dysbiosis of the microbiome. Our approach focused on host proteins involved in microbiota management. D, Prospective enrollment of premature infants with NEC and other confirmed infections. E, Workflow of stool sample preparation was optimized for assay reproducibility and standardization. GI indicates gastrointestinal; IAP, intestinal alkaline phosphatase.

There have been many efforts to discover a molecular diagnostic biomarker for NEC (Figure 1A). Despite the publication of more than 2500 prior biomarker studies, meta-analyses have failed to identify an optimal NEC biomarker for routine clinical use.^9,10,11^ The design and power of these studies raise concern: fewer than 30 articles in each decade of analysis were deemed appropriate for meta-analysis. The focus on inflammation and repair proteins in these studies is problematic (Figure 1B). Late-stage disease with systemic inflammatory damage is not ideal for biomarker evaluation because no period of disease reversibility can be defined.^12^ Furthermore, proteins involved in inflammation have limited positive predictive value because sepsis is a comorbidity in 35% to 60% of NEC cases.^13,14,15,16,17^

Necrotizing enterocolitis has been argued to be the antecedent of some cases of late-onset neonatal sepsis (LOS). Neonates, particularly very low-birth-weight infants, are susceptible to sepsis owing to prolonged hospitalizations, invasive instrumentation, underdeveloped innate immunity, and altered immunological responses. The latter 2 physiological states, coupled with an immature intestinal barrier function, can give rise to NEC.^18,19^ From both epidemiological and clinical standpoints, sepsis can confound the use of inflammation proteins as a biomarker for NEC. Sepsis and NEC require careful differential diagnosis, as both may be lethal if not diagnosed and treated appropriately.

Our study evaluated the use of intestinal alkaline phosphatase (IAP) as a diagnostic biomarker for NEC. Recent findings indicate that NEC is preceded and accompanied by changes in gut microbiota (Figure 1C) and that it is associated with host immune pathways responsible for intestinal inflammation.^19,20^ Intestinal alkaline phosphatase detoxifies the surface lipopolysaccharide (LPS) of harmful bacteria by cleaving inorganic phosphate. A component of gram-negative bacterial cell walls, LPS is a potent inducer of innate immune signaling through toll-like receptor 4. Robust IAP function neutralizes the LPS signal, prevents inappropriate proinflammatory signal cascades in the gut, and contributes to beneficial microbiota maturation.

Because IAP activity precedes the initiation of signaling cascades that trigger inflammation, we evaluated the abundance and enzyme activity of IAP shed in stool as measures of the pathobiological need and ability to maintain host-microbiota homeostasis, respectively. A multicenter, prospective diagnostic study was conducted to assess the association of 2 IAP biochemical measures with disease severity. As a common core protein in the human stool proteome,^21^ IAP is ideal for noninvasive testing. Content of IAP in stool is expected to increase from released membrane vesicles loaded with IAP if there were risk of bacterial-induced inflammation.^22,23^

## Methods

### Study Design and Participants

This study was approved by the Louisiana State University School of Medicine and Washington University School of Medicine in St Louis institutional review board offices. This diagnostic study followed the Standards for Reporting of Diagnostic Accuracy (STARD) 2015 reporting guideline^24,25^ for full reporting. During a 3-year period (May 2015 to November 2018), preterm infants born younger than 37 weeks gestational age with a birth weight less than 1500 grams were enrolled at Children’s Hospital of New Orleans (n = 29; New Orleans, Louisiana) and Touro Infirmary Hospital (n = 68; New Orleans, Louisiana). Preterm infants born younger than 37 weeks gestational age were enrolled at St Louis Children’s Hospital (n = 39; St Louis, Missouri). Written informed consent of study participants was obtained from a parent or guardian. All infants were sought for study inclusion, thereby forming a consecutive sampling series.

### Deidentified Clinical Data

Clinical data, which included gestational age, birth weight, Apgar scores, delivery type, race/ethnicity, sex, and disposition (ie, death, discharge, or transfer to another facility), were extracted from medical records every 3 months. Of these, only race/ethnicity was defined by a parent. In-hospital data included feeding, antibiotic treatment, laboratory and radiology results, and surgical notes. Clinical findings of NEC (modified Bell stage 1-3), sepsis, and other confirmed non–GI tract infections were reviewed by attending physicians.

### Disease Definitions

Different definitions of NEC have been suggested.^26,27,28,29^ For this study, 2 categories of NEC, derived from clinical documentation, were used (eTable 1 in the Supplement). Radiological signs were defining criteria for our NEC categories; abdominal signs and clinical and laboratory findings were secondary criteria. Suspected NEC was defined as concern for disease based on abnormal clinical and laboratory findings without evidence of pneumatosis intestinalis or portal venous gas on abdominal radiographic images. Severe NEC was defined by radiologic evidence of pneumatosis intestinalis and/or portal venous gas. Patients diagnosed with spontaneous intestinal perforation (SIP) were excluded from the study (eTable 2 in the Supplement).

Diagnosis of neonatal LOS required the appearance of abnormal clinical findings at least 72 hours after birth and blood cultures positive for bacteria not considered a contaminant^30,31^ (eTable 3 in the Supplement). Infants with other confirmed non–GI tract infections had clinical findings with bacterial, viral, or fungal infections identified in body fluids other than blood. The summary of cohorts and diagnoses of NEC, SIP, sepsis, and non–GI tract infections are provided in eTable 4 to eTable 11 in the Supplement.

### Sample Collection and Extraction of Soluble Gut Lumen Contents

A simple protocol for stool handling was developed for evaluation of IAP processes in the gut lumen (eMethods in the Supplement). After written parental consent was obtained, samples were collected biweekly from infant diapers and stored in a 4 °C specimen refrigerator at hospital sites until transport to the laboratory. On receipt, stool samples were prepared for luminal content analyses, and a 200 mg/mL slurry was made with molecular grade water in a sterile microfuge tube. Following vortexing and centrifugation, the supernatant was collected, aliquoted, and banked at −80 °C (Figure 1E).

### Protein Concentration

Total protein concentration in the stool supernatant was determined by Bradford assay (ThermoFisher Scientific). Total protein was used to standardize biochemical activity measurements and protein load for quantitative IAP abundance via immunoblot analyses. Protein concentration measurement was reproducible and accurate between replicates and different operators^32^ (eFigure 1, eTable 12, and eMethods in the Supplement).

### Fecal IAP Catalytic Activity

Alkaline phosphatase activity was measured with use of 4-methylumbelliferyl phosphate (Abcam) substrate in the presence and absence of L-phenylalanine, an inhibitor of IAP.^33,34^ Relative fluorescence units at 360/440 nm were measured in a multiwell format on either a Spectra Max M2e or i3x spectrophotometer (Molecular Devices). Total alkaline phosphatase catalysis and 10 mM phenylalanine-inhibited alkaline phosphatase catalysis were measured in triplicate and averaged. Reported IAP activity represents the difference between these 2 averages. We reported IAP activity as 1 μmol of 4-methylumbelliferyl phosphate hydrolysis per minute per gram of total protein in stool supernatant at pH 10.0; individual measurements are in eTable 13, eTable 14, and eTable 15 in the Supplement. Intestinal alkaline phosphatase activity was reproducible between users and on different days (eFigure 1, eTable 12, and eMethods in the Supplement).

### Denaturing Gel Electrophoresis and Immunoblot

We determined IAP abundance using affinity-based methods and reported abundance relative to IAP measured in control human small intestine lysate of equivalent protein load. Duplicate, precast denaturing SDS-PAGE gels (ThermoFisher Scientific) were used to visualize proteins prior to immunoblotting detection of IAP; 5 μg total protein was run per sample. To confirm relative protein abundance^35,36,37^ of IAP, 2 loading controls were run on each gel. The positive control was a single lot of human small intestinal lysate (Abcam). Purified bovine alkaline phosphatase from intestinal mucosa (Sigma) was our negative control. Immunoblotting was performed using traditional or iBlot-iBind methods (ThermoFisher Scientific).^38,39,40^ The amount of IAP in clinical samples was reported as a percent of the detected protein in an immunoblot relative to the difference in densitometric pixel count in a fixed area (Amersham Imager 600; GE Healthcare) that captured the IAP signal in the positive and negative controls (eMethods in the Supplement). A single lot of primary antibody against human IAP, which did not cross-react with other human alkaline phosphatase or negative control proteins (eFigure 1C in the Supplement), and a single lot of horseradish peroxidase–conjugated secondary antibody (Abcam) were used for all analyses. Determinations of IAP content were linear up to 1 μg small intestinal lysate (eFigure 1D in the Supplement).

### Statistical Analysis

Sample size and power calculations for planning this study were based on preliminary data acquired from 6 NEC and 12 non-NEC stool samples from premature infants. From this initial evaluation of the effect size of IAP abundance and dysfunction, it was determined that at least 12 patients with NEC were needed to demonstrate significant difference (ie, with a 5% CI, 2-sided, 2-sample *t* test, and 95% power).^41^ With an assumed event rate of dichotomous outcome of 10% (ie, percent preterm infants born ≤1.5 kg who develop NEC) and a 10% attrition rate, our target enrollment was 130 very low-birth-weight infants.

Associations between inflammatory disease (NEC and non–GI tract infections), neonatal variables, and hospital course were evaluated (Table 1 and Table 2). When characteristics or conditions were considered antecedent or concurrent with disease modality, adjusted associations were evaluated using logistic regression models fit to the binary disease outcome. If the outcome was continuous (eg, the association of sepsis with the number of days in hospital), adjusted associations were evaluated by linear regression; an analysis of variance, *t* test, or Kruskal-Wallis and Wilcoxon test was adopted, depending on the validation of data normality. For unadjusted comparisons or very small counts, statistical significance was determined by χ^2^ or Fisher exact tests. All analyses were completed using SAS version 9.4 (SAS Institute).

**Table 1.** Clinical Characteristics of Patients With Severe NEC, Suspected NEC, or No NEC

Characteristic	No. (%)			*P* Value^a^
	Severe NEC (n = 25)	Suspected NEC (n = 19)	No NEC (n = 92)	
Birth weight, median (IQR), g	855 (700-1380)	940 (790-1190)	1100 (845-1380)	.28
Gestational age at birth, median (IQR), wk	27.6 (24.7-31.1)	28.0 (26.0-29.4)	28.7 (26.4-31.6)	.48
Sex				
Male	13 (52)	12 (63)	42 (46)	.39
Female	12 (48)	7 (37)	49 (54)	
Race/ethnicity				
African American	10 (40)	14 (74)	63 (69)	.08
Caucasian	13 (52)	5 (26)	24 (26)	
Hispanic	2 (8)	0	2 (2)	
Other^b^	0	0	2 (2)	
Cesarean delivery	20 (80)	14 (74)	62 (68)	.51
Apgar scores, median (IQR)				
1 min	5 (2-7)	5 (1-8)	5.5 (3-8)	.75
5 min	7 (5-9)	8 (4-8)	8 (6-9)	.28
First NEC episode, median (IQR)				
PCA, wk	33.9 (31.0-35.7)	29.4 (28.4-30.9)	NA	.02
Day of life, d	21 (10-52)	13 (7-27)	NA	.004
Weight, g	1620 (1110-2050)	1015 (860-1377)	NA	<.001
Repeated NEC episodes	3 (12)	6 (32)	NA	.14
PCA at first stool analyzed, median (IQR), wk	29.9 (27.9-34.3)	29.1 (27.3-30.7)	31.4 (29.0-33.7)	.09
Died before discharge	3 (12)	1 (5)	5 (5.5)	.44
Sepsis comorbidity				
Diagnosed	9 (35)	4 (21)	13 (14)	.24
Suspected	2 (8)	2 (10)	10 (11)	
Length of antibiotic treatment, median (IQR), % of d in NICU	17 (9-25)	12 (7-20)	7 (1-8)	.006
Inotrope exposure	5 (20)	3 (16)	11 (12)	.53
Blood transfusions, median (IQR), No.				
Before NEC	1 (0-4)	1 (0-3)	NA	.77
Total	5 (2-11)	5 (1-6)	0 (0-3)	<.001
NPO, median (IQR), d	10 (8-24)	7 (4-13)	2 (1-4)	<.001
Exposure to human milk, %				
0	4 (16)	3 (16)	8 (9)	.31
>0 to <10	4 (16)	0	10 (11)	
10-50	4 (16)	2 (11)	19 (21)	
51-99	3 (12)	6 (32)	27 (30)	
100	10 (40)	8 (42)	27 (30)	

Abbreviations: IQR, interquartile range; NA, not applicable; NEC, necrotizing enterocolitis; NICU, neonatal intensive care unit; NPO, nil per os; PCA, postconceptual age.

^a^ Using the appropriate method (analysis of variance, Kruskal-Wallis, or Fisher exact test) to compare differences among groups, *P* < .05 indicated that there were statistically significant differences among the 3 infant populations.

^b^ Identified as more than 1 race by parents.

**Table 2.** Clinical Characteristics of Patients With Other Confirmed Infections

Characteristic	No. (%)			*P* Value^a^
	Late-Onset Neonatal Sepsis (n = 26)	Other Non–GI Tract Infection (n = 14)	No Other Infection(n = 96)	
Birth weight, median (IQR), g	790 (670-1010)	830 (700-915)	1165 (912.5-1410)	<.001
Gestational age at birth, median (IQR), wk	25.9 (25-29.7)	26.4 (25-27.1)	29.3 (26.9-32.2)	<.001
Sex				
Male	11 (42.3)	6 (42.9)	50 (52.1)	.63
Female	15 (57.7)	8 (57.1)	46 (47.9)	
Race/ethnicity				
African American	15 (57.7)	12 (85.7)	61 (63.5)	.06
Caucasian	8 (30.8)	1 (7.1)	33 (34.4)	
Hispanic	2 (7.7)	1 (7.1)	2 (2.1)	
Other^b^	1 (3.8)	0	1 (1.0)	
Cesarean delivery	19 (73.1)	10 (71.4)	68 (70.8)	>.99
Apgar scores, median (IQR)				
1 min	5 (2-7)	3 (2-4)	6 (3-8)	.04
5 min	7 (5-8)	6 (5-7)	8 (7-9)	.002
Infection episode closest in time to NEC, median (IQR)				
PCA, wk	31.1 (28.4-34.0)	30.6 (28.7-32.3)	NA	.35
Day of life, d	22 (13-46)	29.5 (23-38)	NA	.43
Weight, g	1140 (950-1700)	1130 (955-1360)	NA	.78
Repeated infection episodes	19 (73.1)	9 (64.2)	NA	.72
PCA at first stool analyzed, median (IQR), wk	29.8 (27.4-31.9)	29.5 (27-31.6)	31 (28.7-33.9)	.06
Died before discharge	3 (11.5)	0	6 (6.3)	.53
NEC comorbidity				
Severe NEC	9 (34.6)	2 (14.3)	15 (15.6)	.24
Suspected NEC	4 (15.4)	2 (14.3)	13 (13.5)	
Length of antibiotic treatment, median (IQR), % of d in NICU	23 (16.7-30)	17 (11-19)	6 (0-14)	<.001
Inotrope exposure	4 (15.4)	6 (42.9)	9 (9.4)	.007
Blood transfusions, median (IQR), No.	5 (2-12)	6.5 (3-11)	0 (0-2.5)	<.001
NPO days, median (IQR), No.	8.5 (6-20)	6.5 (4-13)	2.5 (1-7)	<.001
Exposure to human milk, %				
0	1 (3.8)	2 (14.3)	12 (12.5)	.20
>0 to <10	3 (11.5)	3 (21.4)	8 (8.2)	
10-50	4 (15.4)	0	21 (21.9)	
51-99	9 (35.5)	6 (42.8)	22 (22.9)	
100	9 (35.5)	3 (21.4)	33 (34.4)	

Abbreviations: GI, gastrointestinal; IQR, interquartile range; NA, not applicable; NEC, necrotizing enterocolitis; NICU, neonatal intensive care unit; NPO, nil per os; PCA, postconceptual age.

^a^ Using the appropriate method (analysis of variance, Kruskal-Wallis, or Fisher exact test) to compare differences among groups, *P* < .05 indicated that there were statistically significant differences among the 3 infant populations.

^b^ Identified as more than 1 race by parents.

Each clinical modality was treated as a binary variable to age-appropriate controls. Differences in medians between NEC and control groups for IAP activity and abundance were tested using Mann-Whitney U test; a 2-tailed *P* < .05 was considered statistically significant in highlighting categorical differences. Potential biomarker efficacy was assessed via sensitivity (true-positive rate) and specificity (true-negative rate) calculation. For each variable of interest, specificity and sensitivity were initially obtained using a simple threshold-based classifier. Receiver operating characteristic curve analysis was used to evaluate sensitivity and specificity of the biomarker for the best discrimination between infant samples with or without disease. The Wilson-Brown method for confidence interval determination was used. These statistical calculations were performed using Prism version 8.1.2 (GraphPad). All figures were generated in Igor Pro version 8.0 (Wavemetric).

## Results

A total of 136 infants were enrolled (68 [50.0%] male infants), with a median (interquartile range [IQR]) birth weight of 1050 (790-1350) g and a median (IQR) gestational age of 28.4 (26.0-30.9) weeks*.* A total of 25 (18.4%) were classified as having severe NEC, 19 (14.0%) were suspected of having NEC, and 92 (66.9%) had no NEC (ie, control) (Figure 1D). Of the infants with severe NEC, 19 events (76.0%) took place between 26 and 35 weeks’ postconceptual age (PCA), and 6 (24.0%) took place between 36 and 40 or more weeks’ PCA. For infants classified with suspected NEC, 16 events (84.2%) took place between 26 and 30 weeks’ PCA, and 3 (15.8%) took place between 31 and 35 weeks’ PCA. Study participants had other forms of confirmed infections besides NEC; 26 (19.1%) were diagnosed with LOS, and 14 (10.3%) had a non–GI tract infection (Figure 1D). An equivalent number of male and female infants were enrolled.

Attrition rate was 11.0% (ie, 15 infants), resulting from enrollment changes, medical changes, or inadequate biospecimen collection (Figure 1D). A total of 6 (4.4%) patients were excluded because of withdrawal of parental consent or death (pulmonary or multiorgan failure not related to NEC) before sample collection. A total of 9 (6.6%) enrollees were removed because of diagnosis of SIP, inadequate stool collection, or no stool collection during the episode of suspected or severe NEC. The number of remaining enrollees was 121.

Demographic data and clinical histories were reviewed after stool analyses (Figure 1E). We compiled 5400 demographic and clinical-course characteristics (Table 1 and Table 2). Potentially confounding variables were cross-tabulated for disease. Postconceptual age and weight were the only pre-event clinical variables associated with NEC (Table 1), supporting postnatal disease development as a consistent risk factor (median [IQR] PCA at first NEC episode: severe NEC, 33.9 [31.0-35.7] weeks; suspected NEC, 29.4 [28.4-30.9] weeks; *P* = .02; median [IQR] weight at first NEC episode: severe NEC, 1620 [1110-2050] g; suspected NEC, 1015 [860-1377] g; *P* < .001).^18^ In contrast, birth weight and gestational age were strongly associated with risk of LOS (median [IQR] birth weight: LOS, 790 [670-1010] g; other non–GI tract infections, 830 [700-915] g; no other non–GI tract infection, 1165 [912.5-1410] g; *P* < .001; median [IQR] gestational age at birth: LOS, 25.9 [25.0-29.7] weeks; other non–GI tract infections, 26.4 [25.0-27.1] weeks; no other non–GI tract infection, 29.3 [26.9-32.2] weeks; *P* < .001) (Table 2).^14^

### Abundance of IAP Protein and IAP Enzyme Activity in Patients With Severe NEC, Suspected NEC, and No NEC

**Figure 2.**
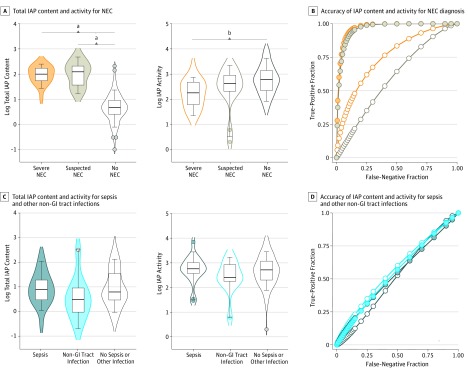
Association of Fecal Intestinal Alkaline Phosphatase (IAP) Content and Activity With Necrotizing Enterocolitis (NEC) and Other Confirmed Infections A, Box and violin plots of fecal abundance and activity of IAP are shown for samples collected at the time of severe (n = 20) and suspected NEC (n = 15). Samples from patients with no NEC (n = 86), age-matched at the time of sample collection for NEC, are also shown. Box plot whiskers mark 9th and 91st percentiles. B, Receiver operating characteristic curves for IAP abundance (filled circles) and activity (open circles) in samples collected during severe (orange) or suspected (brown) NEC. C, Box and violin plots of fecal abundance and activity of IAP are shown for samples collected during sepsis (n = 18), other non–gastrointestinal (GI) tract infection (n = 10), and age-matched control patients (n = 91). Box plot whiskers mark 9th and 91st percentiles. D, Receiver operating characteristic curves of IAP abundance (filled circles) and activity (open circles) in samples collected during sepsis (dark blue) and other non–GI tract infections (light blue). ^a^*P* < .001 ^b^*P* = .005

Infants with NEC had high relative IAP content in their stool samples at the time of clinical diagnosis (Figure 2A). Samples collected at the time of severe NEC had a median (IQR) IAP content of 99.0% (51.0%-187.8%) (95% CI, 54.0%-163.0%), whereas control samples had a median (IQR) IAP content of 4.8% (2.4%-9.8%) (95% CI, 3.4%-5.9%). Increased fecal IAP protein was associated not only with severe NEC but also suspected disease. Stool samples collected at the time of NEC suspicion had a median (IQR) IAP content of 123.0% (31.0%-224.0%) (95% CI, 31.0%-224.0%) (Figure 2A). The median IAP abundance in stool at the time of severe NEC and suspected NEC was increased 20-fold compared with stool collected from age-matched controls with no NEC.

Activity of IAP in samples collected during episodes of suspected and severe NEC was significantly lower compared with samples from infants who did not have NEC (Figure 2A). However, different levels of IAP enzyme dysfunction were found between patients with suspected and severe NEC. Samples at the time of severe NEC had a median (IQR) IAP activity of 183 (56-507) μmol/min/g (95% CI, 63-478 μmol/min/g) of stool protein. Samples at the time of suspected NEC had a median (IQR) IAP activity of 355 (172-608) μmol/min/g (95% CI, 172-608 μmol/min/g) of stool protein, and IAP activity in PCA-matched control samples had a median (IQR) of 613 (210-1465) μmol/min/g (95% CI, 386-723 μmol/min/g) of stool protein. Thus, infants with severe NEC had only a quarter of the ability to modulate aberrant bacterial colonization as their counterparts with suspected or no NEC, suggesting a dysfunction in host-microbial crosstalk.

### Sensitivity, Specificity, and Positive Predictive Value of Fecal IAP Measures

Accuracy, or area under the curve, of the single biochemical measure of IAP was evaluated using a receiver operating characteristic curve, a common tool used to calculate clinical prediction rules (Figure 2B). Mean (SE) accuracy using IAP content as a marker for severe NEC was 0.97 (0.02) (95% CI, 0.93-1.00; *P* < .001), and mean (SE) accuracy using IAP activity as a marker for severe NEC was 0.76 (0.06) (95% CI, 0.64-0.86; *P* < .001). Similar mean (SE) accuracy values of 0.97 (0.02) (95% CI, 0.93-1.00; *P* < .001) for IAP content and 0.62 (0.07) (95% CI, 0.48-0.77; *P* = .13) for IAP activity were obtained for suspected NEC.

In contrast, IAP content and activity lacked accuracy in the diagnosis of sepsis and other non–GI tract infections (Figure 2C). There was negligible IAP shed in stool collected at the time of clinically defined sepsis (median [IQR], 6.5% [2.2%-23.1%]; 95% CI, 2.2%-19.8%), other non–GI tract infections (median [IQR], 3.1% [0.8%-10.9%]; 95% CI, 0.6%-15.2%), and controls (median [IQR], 6.2% [2.7%-40.0%]; 95% CI, 4.6%-11.0%). Enzymatic ability of IAP did not differ statistically between samples collected from these 3 cohorts (Figure 2C); median (IQR) activity for sepsis was 575 (338-1122) μmol/min/g (95% CI, 355-1073 μmol/min/g) of stool protein, for other non–GI tract infections, 319 (207-961) μmol/min/g (95% CI, 172-1193 μmol/min/g) of stool protein, and, for the control group, 519 (180-1243) μmol/min/g (95% CI, 350-695 μmol/min/g) of stool protein. Area under the receiver operating characteristic curves showed that use of fecal IAP content or activity would randomly assign culture-confirmed bacterial sepsis and other non–GI infection as positives or negatives for these inflammatory conditions (Figure 2D). Mean (SE) accuracy scores for IAP content were 0.52 (0.07) (95% CI, 0.38-0.66; *P* = .75) at the time of sepsis and 0.58 (0.08) (95% CI, 0.42-0.75; *P* = .06) at the time of other non–GI infection. Mean (SE) accuracy scores for IAP activity were 0.52 (0.07) (95% CI, 0.39-0.67; *P* = .68) at the time of sepsis and 0.57 (0.08) (95% CI, 0.39-0.69; *P* = .66) at the time of other non–GI infection.

## Discussion

Necrotizing enterocolitis and LOS in neonates have exaggerated inflammatory responses and a number of common attributes. Differential diagnosis is complicated by their overlapping presentations, diagnostic tools with limited sensitivity, and even their evolving definitions.^42,43^ Current criterion standards are abdominal radiography for NEC and positive blood culture for sepsis. Yet both standards suffer from low sensitivity and the possibility of causing harm from excessive radiation exposure or blood sampling. Lastly, outcome reports are problematic: interpretations of subtle radiological findings are subjective and may vary, whereas culture results may take up to 48 to 72 hours.

There have been numerous attempts to identify candidate markers of gut injury that discriminate NEC from other inflammatory conditions.^44,45,46,47,48^ Animal NEC models suggest that the immune dysregulation and microbial dysbiosis associated with severe NEC are tandem host-bacterial missteps owing to excessive toll-like receptor 4 signaling in response to bacterial LPS.^19,49,50,51,52^ The majority of candidate NEC biomarkers are proteins further downstream from the initial host signaling steps. Elevations in platelet activating factor,^3,53^ inter-α inhibitor protein,^54^ calprotectin, claudin,^48^ intestinal fatty acid binding protein,^55^ and C-reactive protein^56^ in plasma have been associated with NEC onset. Taken together, current literature points toward the idea that diagnosis of advanced NEC is a clinical descriptor of terminal-stage pathologic processes,^29,57^ suggesting that an NEC biomarker may always be confounded by sepsis.

Our study challenged these theories. Biomarkers, such as calprotectin, are reliable indicators of intestinal inflammation in general but provide no understanding of the dominant inflammatory pathways at work in the intestinal mucosa of a patient. Our study required prospective inclusion of infants with NEC and concurrently tested healthy and unhealthy controls with several inflammatory conditions in the neonatal intensive care unit. Under these real-life conditions, estimates of biomarker reliability more accurately reflected potential performance in clinical application. Examination of proteins involved in organ-specific modulation of microbiota homeostasis and response distinguished NEC from other forms of inflammation. As such, IAP is the first candidate diagnostic biomarker, unique in its high positive predictive value for NEC. Importantly, IAP is associated with NEC and not associated with sepsis or other non–GI tract infections.

Using a protein that is an established antecedent to inflammation, induced by LPS, as a biomarker has support from prior studies. There are several models of IAP activation in gut dysbiosis: exosomes, increased gut permeability, and/or intestinal epithelial injury. It has not been clarified whether the bacterial translocation across the gut epithelium that can give rise to LOS is a native outcome from altered gut epithelial permeability or a result of gut barrier deterioration. Our IAP study does not address whether there is deterioration of the gut endothelium in NEC or sepsis. However, detection of IAP in such high abundance in our stool samples during NEC episodes suggested that there is active regulation of lipid vesicle secretion into the gut lumen during active NEC disease; such secretion of IAP is not detectable in stool during LOS. This investigation does not support the idea that NEC shares the same pathobiological mechanism as neonatal sepsis.

The IAP biomarker is associated with disease severity; IAP biochemistry differentiates advanced NEC, flagged by portal venous gas or pneumatosis intestinalis, from suspected disease, for which there are no reliably observable signs by radiology. Our results also showed that this classification of NEC suspicion is supported as an explicit disease state. Our approach differed from other candidate biomarker studies. This work diverges not only by the target protein of interest but also by our use of a disease severity catalog, biospecimen choice, and molecular method of detection. We were able to segregate NEC suspicion from severe cases of NEC. There has been great effort to identify commonalities in clinical criteria to define severe NEC. Very few reports on NEC suspicion are published because of the absence of a molecular diagnostic test and lack of definition consensus. This study showed that suspected and severe NEC were associated with the active release of IAP in infant stool. It also demonstrated that there were clear differences in IAP function in these 2 disease categories. Advanced NEC was associated with severe biochemical dysfunction of host IAP, whereas suspected NEC has only partial loss of IAP enzyme activity. In contrast, C-reactive protein and other biomarkers are not associated with Bell staging,^11^ and importantly, the values do not significantly vary between suspected and severe NEC.

Our findings did differ from the other studies evaluating IAP as a biomarker for NEC. Our research report used not 1 but 2 measures to evaluate IAP biochemistry in patient samples, as follows: (1) immunoblotting to quantify its relative abundance in comparison with the amount of IAP found in human small intestine and (2) enzymatic activity to identify whether the protein is functional and capable of modulating microbial dysbiosis. Both approaches are necessary to distinguish disease pathways and differences between individuals. Serological tests^58^ of alkaline phosphatase as an NEC biomarker reported that the amount of IAP in blood was increased in infants with NEC compared with controls, suggesting that IAP may play a role in NEC pathogenesis. Serum is not an ideal sampling source, as 4 different alkaline phosphatases are present, and their relative levels in serum are known to change during gestation^59^ (eFigure 1 and eFigure 2 in the Supplement). Although prior conclusions drawn^58^ support our findings, sole use of denaturing protein gels cannot provide equivalent evidence that IAP was identified nor is it capable of quantifying the amount of alkaline phosphatase in general.

### Limitations

Limitations to this study include sporadic stooling patterns associated with prematurity, which did not permit standardized collection times. Furthermore, not all NEC samples were obtained, as there is often decreased stooling with acute illness. However, given the noninvasive nature of stool collection, this process offers clear clinical advantages over serological testing that can lead to iatrogenic blood loss in infants.

## Conclusions

In conclusion, the results of this study indicated that the measurement of IAP dysfunction in stool is a biomarker for NEC with better sensitivity and specificity than other candidates previously reported in the literature. Although promising, use of fecal IAP as a biomarker should be considered an adjunct in establishing the diagnosis of severe NEC, monitoring disease progression, and surveilling high-risk infant groups. Normative data across different PCAs are needed for appropriate design and analysis of future biomarker studies to determine whether fecal IAP can serve as a diagnostic proxy at the molecular level. The clinical potential of this noninvasive tool lies in its ability to identify infants most at risk of developing NEC, to facilitate management of feeding and antibiotic regimens, and to monitor response to treatment.

## Notes

Supplement. **eMethods.** Clinical Data, Disease Definitions, and Biospecimen Collection and Analysis **eTable 1. **Study Criteria Used to Classify Diagnosis and Suspicion of Neonatal Necrotizing Enterocolitis **eTable 2. **Study Criteria Used for Focal or Spontaneous Intestinal Perforation **eTable 3. **Study Criteria Used to Define Pathogenic Infection Outside the Gastrointestinal Tract **eTable 4. **Summary of NEC Cohorts at Different Clinical Sites **eTable 5. **List of 25 Radiologically Confirmed (Severe) Cases of Necrotizing Enterocolitis Enrolled **eTable 6. **List of 19 Suspected Necrotizing Enterocolitis Cases Enrolled **eTable 7. **List of 3 Enrolled Infants With Spontaneous Intestinal Perforation (SIP) and Necrotizing Enterocolitis **eTable 8. **List of 86 Enrolled Infants Who Were Neither Clinically Diagnosed With nor Suspected of Having Necrotizing Enterocolitis **eTable 9. **Summary of Sepsis and Other Non–GI Tract Infection Cohorts at Different Clinical Sites **eTable 10. **List of All 26 Late-Onset Neonatal Sepsis Cases Enrolled **eTable 11. **List of All 14 Cases of Confirmed, Non–GI Tract Infections in Urine, Bone, or Trachea**** **eTable 12. **Accuracy and Reproducibility of In Vitro Measurements of Gut Lumen Content **eTable 13. **IAP Measurements From 20 Stool Samples at the Time of Severe Necrotizing Enterocolitis **eTable 14. **IAP Measurements From 15 Stool Samples at the Time of Necrotizing Enterocolitis Suspicion **eTable 15. **IAP Measurements From 86 Enrolled Infants Who Were Neither Clinically Diagnosed With nor Suspected of Having Necrotizing Enterocolitis **eTable 16. **Proteins Identified in Preterm Gut Lumen (N = 635) **eFigure 1. **Control Experiments Demonstrated Operator Reproducibility, Antibody Reagent Specificity, and Biospecimen Specificity **eFigure 2. **Sequence Alignment of 4 Human Alkaline Phosphatases and Calf Intestinal Alkaline Phosphatase **eReferences**
