# Supplementary material for: Association of Intestinal Alkaline Phosphatase With Necrotizing Enterocolitis Among Premature Infants
Source: JAMA Netw Open. 2019 Nov 8;2(11):e1914996. doi: 10.1001/jamanetworkopen.2019.14996 (PMC6902776; doi:10.1001/jamanetworkopen.2019.14996)
Supplement: Supplement. — eMethods. Clinical Data, Disease Definitions, and Biospecimen Collection and Analysis eTable 1. Study Criteria Used to Classify Diagnosis and Suspicion of Neonatal Necrotizing Enterocolitis eTable 2. Study Criteria Used for Focal or Spontaneous Intestinal Perforation eTable 3. Study Criteria Used to Define Pathogenic Infection Outside the Gastrointestinal Tract eTable 4. Summary of NEC Cohorts at Different Clinical Sites eTable 5. List of 25 Radiologically Confirmed (Severe) Cases of Necrotizing Enterocolitis Enrolled eTable 6. List of 19 Suspected Necrotizing Enterocolitis Cases Enrolled eTable 7. List of 3 Enrolled Infants With Spontaneous Intestinal Perforation (SIP) and Necrotizing Enterocolitis eTable 8. List of 86 Enrolled Infants Who Were Neither Clinically Diagnosed With nor Suspected of Having Necrotizing Enterocolitis eTable 9. Summary of Sepsis and Other Non–GI Tract Infection Cohorts at Different Clinical Sites eTable 10. List of All 26 Late-Onset Neonatal Sepsis Cases Enrolled eTable 11. List of All 14 Cases of Confirmed, Non–GI Tract Infections in Urine, Bone, or Trachea eTable 12. Accuracy and Reproducibility of In Vitro Measurements of Gut Lumen Content eTable 13. IAP Measurements From 20 Stool Samples at the Time of Severe Necrotizing Enterocolitis eTable 14. IAP Measurements From 15 Stool Samples at the Time of Necrotizing Enterocolitis Suspicion eTable 15. IAP Measurements From 86 Enrolled Infants Who Were Neither Clinically Diagnosed With nor Suspected of Having Necrotizing Enterocolitis eTable 16. Proteins Identified in Preterm Gut Lumen (N = 635) eFigure 1. Control Experiments Demonstrated Operator Reproducibility, Antibody Reagent Specificity, and Biospecimen Specificity eFigure 2. Sequence Alignment of 4 Human Alkaline Phosphatases and Calf Intestinal Alkaline Phosphatase eReferences [file jamanetwopen-2-e1914996-s001.pdf]

Supplementary Online Content

Heath M, Buckley R, Gerber Z, et al. Association of intestinal alkaline phosphatase with necrotizing enterocolitis among premature infants. *JAMA Netw Open*. 2019;2(11):e1914996. doi:10.1001/jamanetworkopen.2019.14996

eMethods. Clinical Data, Disease Definitions, and Biospecimen Collection and Analysis
eTable 1. Study Criteria Used to Classify Diagnosis and Suspicion of Neonatal Necrotizing Enterocolitis
eTable 2. Study Criteria Used for Focal or Spontaneous Intestinal Perforation
eTable 3. Study Criteria Used to Define Pathogenic Infection Outside the Gastrointestinal Tract
eTable 4. Summary of NEC Cohorts at Different Clinical Sites
eTable 5. List of 25 Radiologically Confirmed (Severe) Cases of Necrotizing Enterocolitis Enrolled
eTable 6. List of 19 Suspected Necrotizing Enterocolitis Cases Enrolled
eTable 7. List of 3 Enrolled Infants With Spontaneous Intestinal Perforation (SIP) and Necrotizing Enterocolitis
eTable 8. List of 86 Enrolled Infants Who Were Neither Clinically Diagnosed With nor Suspected of Having Necrotizing Enterocolitis
eTable 9. Summary of Sepsis and Other Non–GI Tract Infection Cohorts at Different Clinical Sites
eTable 10. List of All 26 Late-Onset Neonatal Sepsis Cases Enrolled
eTable 11. List of All 14 Cases of Confirmed, Non–GI Tract Infections in Urine, Bone, or Trachea
eTable 12. Accuracy and Reproducibility of In Vitro Measurements of Gut Lumen Content
eTable 13. IAP Measurements From 20 Stool Samples at the Time of Severe Necrotizing Enterocolitis
eTable 14. IAP Measurements From 15 Stool Samples at the Time of Necrotizing Enterocolitis Suspicion
eTable 15. IAP Measurements From 86 Enrolled Infants Who Were Neither Clinically Diagnosed With nor Suspected of Having Necrotizing Enterocolitis
eTable 16. Proteins Identified in Preterm Gut Lumen (N = 635)
eFigure 1. Control Experiments Demonstrated Operator Reproducibility, Antibody Reagent Specificity, and Biospecimen Specificity
eFigure 2. Sequence Alignment of 4 Human Alkaline Phosphatases and Calf Intestinal Alkaline Phosphatase
eReferences

This supplementary material has been provided by the authors to give readers additional information about their work.

© 2019 Heath M et al. *JAMA Network Open.*

eMETHODS

CLINICAL DATA: To protect confidentiality and anonymity, each enrolled patient was provided a code, which allowed for research tracking and removed any clues to the individual's identity. Every three months, patient records were evaluated to determine clinical correlatives. Clinical data were extracted from medical records into a relational clinical database. Demographic information and initial clinical data included gestational age, birth weight, Apgar scores, delivery type, race, gender, and final outcome (death, discharge, or transfer). Lastly, a second set of clinical information was obtained: antibiotic use, diet, serum AP, radiology reports, length of stay in NICU, surgery, and mortality. Human milk exposure is calculated as the mean percent of feeding from human milk as a function of the total days that the subjects were in the study. For NEC cases, only pre-event exposures were considered for human milk. Antibiotic exposures were considered in aggregate; antibiotics were always parentally administered to subjects. Percent of days of age on antibiotics is related to the number of days that the subjects were in the study. For NEC cases, only pre-event exposures were considered for antibiotics.

DISEASE DEFINITIONS: Clinical findings of NEC diagnosis, NEC suspicion, sepsis, and other confirmed non-GI infections were identified from review of clinical documentation. The research definition of NEC did not always align with the clinical diagnosis of the patient. For this study, NEC and suspected NEC were physician-directed clinical diagnoses in which radiologic signs were the defining criteria and abdominal signs, clinical findings, and lab findings further confirmed diagnosis (eTable 1). **NEC suspicion** (eTable 1) was defined as an infant with concern for early disease based on clinical and laboratory abnormalities without evidence of *pneumatosis intestinalis* on radiography. Instead, infants suspected of NEC exhibited one or more radiologic signs including mild intestinal dilation, mild intestinal ileus, thickened bowel walls, or paucity/absence of bowel gas. One or more of the clinical or abdominal signs and symptoms in addition to one or more laboratory finding(s) including decreased platelets, decreased or increased white blood cells, decreased absolute neutrophil count, increase in number of immature neutrophils, heme-positive stool, metabolic acidosis) were also required. Clinical and abdominal signs and symptoms included bilious aspirates, emesis, bloody stool, feeding intolerance, increased pre-feed gastric residual volume, increased apneas and/or bradycardias, temperature instability, lethargy, generalized clinically ill-appearing, mild to moderate abdominal distention and, abdominal wall discoloration. **Severe NEC** (eTable 1) was defined by radiological evidence of *pneumatosis intestinalis* and/or portal venous gas or pathological findings on surgical or postmortem intestinal samples. Pneumoperitoneum, which is free intrabdominal air resulting from a perforation, was considered NEC when accompanied by evidence of pneumatosis intestinalis on radiography, as well as abdominal signs found in definite NEC. Additional signs included moderate to severe abdominal distention and/or abdominal tenderness and/or hypoactivity/absence of bowel sounds and/or abdominal wall discoloration, abdominal cellulitis, fixed right lower quadrant abdominal mass, and or signs of peritonitis. NEC diagnosis was categorized by at least one senior clinician and two additional senior research clinicians by case review, note review, x-ray and operative findings.

Although clinical presentation and medical management is similar to that of NEC, patients with **spontaneous intestinal perforation (SIP)**, which is thought to be a different disease altogether (eTable 2), were excluded from the study. Key differences distinguishing SIP from NEC include absence of pneumatosis intestinalis on abdominal radiography, an earlier onset of symptoms, focal hemorrhagic intestinal necrosis (rather than coagulative necrosis characteristic of NEC) on pathologic specimen, and an overall, more benign clinical course, both preceding and following diagnosis. Overlapping medical management of both SIP and NEC processes include cessation of enteral feeds, gastric decompression, intravenous antibiotics, and peritoneal drainage if indicated.[8,9]

Diagnosis of **neonatal sepsis** is variable and complicated by the usage of biomarkers with overall low sensitivity, such as alterations in white blood cell count indices, low absolute neutrophil counts, high immature-to-total (I:T) neutrophil ratio, and elevated serum C-reactive protein levels.[10] Positive blood cultures at 3 or more days of age for late-onset septicemia is considered the gold standard for diagnosis of neonatal sepsis. However, cultures are frequently negative, likely related to low inoculated blood volume that may not fully represent a true bacteremia and exposure to prenatal antibiotics that may suppress bacterial growth.[11-13]

In this study, infants diagnosed with sepsis include only those with laboratory and clinical findings that are confirmed after seventy-two hours of age (eTable 3). Laboratory findings included blood culture or non-culture microbial testing that confirmed the presence of bacteria in blood that was not considered a contaminant.[14,15] Clinical findings that were used to support the diagnosis of sepsis included a range of criteria from temperature instability and respiratory distress to abnormal perfusion, bleeding issues, and unexplained jaundice.

In addition, infants with **other confirmed non-GI infections** and infants who were infection-negative were classified and documented in this study (eTable 3). Other confirmed infections were those confirmed bacterial, viral or fungal infections identified in normally sterile body fluids. Clinical findings were similar to those diagnosed with sepsis. An infection-negative classification was comprised of infants with suspected, but not confirmed, infections and infants not suspected of any infections for which no laboratory tests were ordered concerning an infection and were asymptomatic. Those suspected of infections had laboratory findings that included, but were not limited to, leukocytosis or leukopenia, elevated immature neutrophil counts, low absolute neutrophil counts, and elevated C-reactive protein and serum alkaline phosphate. Clinical findings for these infants were identical to those with confirmed sepsis diagnosis.

BIOSPECIMEN COLLECTION: Stool samples were collected serially from disposed diapers of study subjects after spontaneous stooling. Initial collection at St. Louis Children's Hospital was only one sample per patient, but shifted to weekly collections per patient. From Children's Hospital of New Orleans and Touro Infirmary Hospital, samples were collected prospectively. Stooling frequency from enrolled infants matched those reported in the literature: for the general pediatric population, the mean bowel frequency is greater than 8 evacuations per week and does not vary in the first 2 years of life.[16] Upon documentation of patient code and date of sample acquisition by nursing staff, stool was stored briefly in hospital specimen 4°C refrigerators, until transport to the lab in cooler boxes. Stool samples from NEC and from non-NEC patients had a pH between 6 and 7 (ColorpHast pH 0-14 indicator strips, Sigma), consistent with reported median pH of 6.64 for stool.[17]

WORKFLOW FOR FECAL MATERIAL FROM GUT LUMEN: It would be expected that specific proteins should shift between insoluble and soluble fractions of stool in a stimulus-dependent manner, such as epithelial release of luminal vesicles from the small intestine microvilli into the lumen. Therefore, stool preparation is a critical factor for accurate quantitative analyses. Stool is a complex matrix: not only is there a variety of biological material (cells from the host infant gut, bacteria, mucin, proteolytic enzymes, etc.), but the different types of biochemical and cellular structures are not represented in equivalent stoichiometry. For example, proteins most biologically relevant to host responses are unlikely to be identified, due to the expected modest representation of host-derived gut proteins in the total stool proteome,[18] partial proteolysis that occurs during gut transit, and a large and ill-defined microbiota proteome. Furthermore, prior gut proteome investigations[19-21] identified or recovered far fewer proteins than typical cell or tissue-based analyses.

To cope with these challenges, we first standardized our assay protocol to the fecal weight of a homogenous patient sample, as it is the most commonly used parameter to evaluate fecal properties:[22-25] Two hundred mg of fresh stool, which typically is 75% water,[17] was weighed (Figure 1E). Sterile, deionized water, free from proteases and DNases water (Sigma Aldrich), was added to make a slurry at 200 mg/mL (weight stool:volume), as buffer composition substantially affects the results of quantitative immunoblotting and activity assay measurements.[26] Then, after quick vortexing, we applied a centrifugation protocol (Figure 1E) that separated intact free-living cells, complexes associated with cell membrane surfaces, and other large particular matter in the pellet.[27,28] The supernatant contained proteins secreted in the gut lumen, on which our analysis is focused. As such, mass spectral analysis confirmed that iAP was found in the supernatant and was readily detectable (eTable 16 and eMethods). We note that cell lysis, for which conditions can have a profound impact on proteins extracted, is not a variable in this study. The supernatant was aliquoted, snap frozen in liquid nitrogen, and stored at -80°C until use.

BIOCHEMICAL MEASUREMENTS OF SECRETED PROTEINS IN GUT LUMEN: Three different protein assays were performed on one aliquot of supernatant (Figure 1E): determination of total protein, enzyme activity assays monitoring alkaline phosphatase catalysis, and detection of intestinal alkaline phosphatase by immunoblot. Intestinal alkaline phosphatase is resistant to intestinal degradation by host digestive enzymes[29] and thermostable.[30] For all three assays, quantitative measurements required standard curves, which were evaluated in replicate on each platform daily. Instrument and pipette calibration were performed every six months by external vendors.

Protein concentration. The concentration of total protein in final stool supernatant was determined by Bradford assay (Coomassie Plus Protein Assay Reagent, Thermo-Scientific) on either a Spectra Max M2e or Spectra Max i3x spectrophotometer (Molecular Devices). Protein standards (bovine serum albumin, Pierce) and patient samples were prepared, using molecular grade water (Millipore) as the diluent. Five-point standard curves were generated for each day of measurement; daily r^2 values ≥0.994 were indicative of linearity of protein abundance measurements. A second measure of analytical validity was the mean error from ideal values of standards used;

eTable 15 illustrates accuracy and precision of three different operators by showing measures of reproducibility and correlation between days and between operators are shown in Table e15.

Fecal iAP catalytic activity. Activity assays are a measure of enzymatic catalysis as a function of time and as a ratio of protein in the stool supernatant. Alkaline phosphatase activity can be measured using a number of different substrates. The substrate employed determines the dynamic range and sensitivity of the enzymatic reaction. AP activity in this work was measured with use of 4-methylumbelliferyl phosphate (MUP) as a fluorescent substrate (Abcam, ab83371) in the presence and absence of L-phenylalanine, an inhibitor of iAP. Use of MUP has technical advantages for our study. Fluorogenic substrates enable catalytic activity of AP to be measured with high sensitivity and accuracy, attributes which are ideal for basic research and biotechnology applications. Second, fluorogenic substrates typically have a detection range that is 100X-1000X greater than chromogenic substrates, for which product precipitates are detected after reduction of tetrazolium salts or production of colored diazo compounds.[31] The 4-MUP substrate has a lower K_m than other native substrates found in human samples.[32] Determination of V_{max} using 4-MUP is pH-independent.[33,34] Lastly, its hydrolysis products do not lead to strong inhibition of alkaline phosphatase,[32] which would lower the measurement range and limit accuracy.

Relative fluorescence units (RFUs) at 360 nm excitation/440 nm emission were measured using a Spectra Max M2e spectrophotometer or Spectra Max i3x (Molecular Devices). Ninety-six-well black optical bottom plates (ThermoScientific) were used. Standards and negative controls were prepared for each plate run. A 100 mM stock of L-phenylalanine (purity >98%; Sigma Aldrich) was freshly prepared in molecular grade water each day of use. A final assay concentration of 10 mM Phe was used to assess inhibition of iAP-specific activity.

Denaturing gel electrophoresis and immunoblot. Our approach was to compare the amount of fecal iAP, which reflects the abundance of protein in the gut lumen, relative to iAP protein in the human intestinal epithelium standard. Supernatant of stool samples were mixed with gel loading buffer (375 mM Tris pH 6.8, 50% (w/v) glycerol, 600 mM dithiothreitol, 420 mM sodium dodecyl sulfate) and boiled for 5 mins. Sample loads per lane were prepared according to total protein.[35-37] A total of 5 µg of total protein was loaded per lane of a denaturing 4-12% iBolt Bis-Tris gel (Novex, Life Technologies). Duplicate gels were run: one was Coomassie-stained to visualize all proteins in each lane and the second was used for immunoblotting.

Akin to ELISA measurements, more than one reference was used for quantitation of relative iAP content in stool samples in our immunoblots. This approach mitigates the danger of single-variable normalization, long recognized in data from microarrays[38] and quantitative PCR.[39] Thus, our positive control was human small intestinal tissue lysate (Abcam). Purified bovine alkaline phosphatase from intestinal mucosa (Sigma Aldrich) was used as a negative control. The primary antibody used was specific for the human intestinal isoform of iAP (eFig. 1C), when evaluated against human small intestine lysate (Abcam; ab29276), purified human placental alkaline phosphatase (ab114268), purified human tissue non-specific alkaline phosphatase (ab114267), and bovine intestinal alkaline phosphatase (Sigma, P5521). Our positive control from a single lot, as well as a single lot of negative control, served as calibrators for all quantitations in the manuscript; both are loaded on each and every gel with patient samples. These two standards were used to define the linear relationship of our anti-iAP signal for patient samples.

Transfer of proteins in gel matrix was performed using one of two techniques: either (1) a semi-dry transfer apparatus (FisherScientific) at a constant 5V for 1 hour or (2) the iBlot 2 (ThermoFisher) dry blotting system at starting at 20V and ending at 25V for a total of 7 min. Western blotting techniques were performed either using traditional methods[40-42] or with use of an iBind system (ThermoFisher). Membranes were either serially blocked in 5% (w/v) nonfat dry milk in 50 mM Tris-HCl pH 7.5, 150 mM NaCl, and 0.1% Tween or with the iBind solution kit (ThermoFisher) reagents. At room temperature, membranes were incubated with primary rabbit polyclonal antibodies against human iAP (Abcam, ab7322) at a 1:13,000 dilution, washed, and incubated with horseradish peroxidase-conjugated goat anti-rabbit secondary antibodies (Abcam, ab6721) at a 1:20,000 dilution.

Bands were quantified on an Amersham Imager 600 (GE Healthcare); its CCD chip and large aperture FujiIon f/0.85 43 mm lens allows for greater sensitivity for low-light applications. The iAP protein in the positive control lane was manually defined. Equivalent areas were quantitated for each lane of the immunoblot, including the negative control and patient samples. The resulting signal for each patient sample was divided by the difference between the positive control and the negative control to give a final percentage of the positive control standard. To account for sample prep, detection scheme, and normalization approach in our hands, a calibration curve of a lysate of human intestinal epithelium (eFig. 1D), our positive control, showed overlap between linear portion of anti-human iAP signal detection and our working range.[43]

Accuracy and reproducibility measurements. Although five different operators performed these assays, examination of replicates showed clear reproducibility (eFigs. 1A and 1B) suggesting that biological signal can be differentiated from noise in these assays.

Accuracy and reliability of each biochemical test used to calculate iAP biomarker sensitivity and specificity were assessed (Table e16). To minimize the impact of batch effects,[44] five independent measurements of known analyte concentrations for each biochemical assay were selected randomly from an eight-month period by three different operators. Accuracy was assessed by comparing the experimental measurement of the analyte and the absolute measure as defined by the manufacturer and reported as a percent of the absolute value (eFig. 1AB). The absolute value of each concentration of alkaline phosphatase used for activity assays was measured on the Tecan Infinite M1000 Pro (personal communication from the supplier; Abcam). Experimental measurement of the analyte was performed on the SpectraMax i3x (Molecular Devices) with a photometric range of 0-0.4 OD and a photometric resolution of 0.001 OD. For the Bradford assay, the extinction coefficient for bovine serum albumin (BSA; 43,824 M^{-1}) and Beer's law equation were used to calculate the absolute value for each dilution of BSA used for the standard curve measured on the SpectraMax i3x. Accuracy was calculated using the equation: accuracy = [(absolute value – measurement value)/absolute value] x 100%. The reliability, or how reproducibly a measurement of an analyte compared to the absolute value of the analyte, was determined by calculating the standard error and reporting the *p*-value. As the *p*-value indicates whether measurements deviate significantly from each other, it can be used to indicate whether inter-operator measurements for calibrators are either statistically similar (p-value <0.05) or are dissimilar (p-value $\geq$0.05). For all measurements of patient samples, dilutions of the sample were performed to ensure the experimental measurement value fell in the middle of the linear range between the highest and lowest analyte concentration used for the standard curve.

MASS SPECTROMETRY: MS1 scans on 0.5 µg/µL processed stool sample, that was subsequently reduced, alkylated, and trypsinized, were performed in a Fusion Tribrid Orbitrap (Thermo Fisher Dionex, Sunnyvale, CA) utilizing a resolution of 240,000, following liquid chromatography separation on a Dionex U3000 HPLC system (Thermo Fisher Dionex, Sunnyvale, CA). The MS2 scans were performed in the Orbitrap using High Energy Collision Dissociation (HCD) setting of 30% and a resolution of 30,000. This was repeated for a total of three technical replicates. Data analysis was performed using Proteome Discoverer 2.2 using SEQUEST HT scoring. The Protein FASTA database was H. sapiens version 2017-07-05. Static modifications included carbamidomethyl on cysteines (=57.021) and dynamic modification of oxidation of methionine (=15.9949). Parent ion tolerance was 10 ppm, fragment mass tolerance was 0.02 Da, and the maximum number of missed cleavages was set to 2. Only high scoring peptides were considered utilizing a false discovery rate (FDR) of 1%.

© 2019 Heath M et al. *JAMA Network Open.*

eTable 1. Study Criteria Used to Classify Diagnosis and Suspicion of Neonatal Necrotizing Enterocolitis

Study classifications result from minimum, common requirements for disease severity definitions.[1,2] Ranked order of study criteria met was (1) radiological signs, (2) abdominal signs, (3) clinical findings, and (4) lab findings; one radiological sign and one criteria from the other groups were identified for each patient classified as NEC.

STUDY CLASSIFICATION	RADIOLOGIC SIGNS	ABDOMINAL SIGNS	CLINICAL FINDINGS	LABORATORY FINDINGS
**SEVERE NEC** *radiologically-confirmed disease*	pneumatosis intestinalis, portal venous gas, pneumoperitoneum, intestinal perforation	moderate to severe abdominal distention, tenderness, and/or discoloration, abdominal cellulitis, hypoactivity or absence of bowel sounds, fixed right lower quadrant abdominal mass, signs of peritonitis	bilious aspirate, emesis, bloody stool, feeding intolerance, increased pre-feed gastric residual volume, increased apneas and/or bradycardias, temperature instability, lethargy, generalized clinically ill- appearing, systemic instability *(severe apnea, bradycardia, shock, DIC, hypotension, respiratory failure)*	coagulopathy, thrombocytopenia, leukocytosis or leukopenia, neutropenia, bandemia, heme-positive stool, metabolic acidosis
**SUSPECTED NEC** *disease not confirmed by radiology*	no pneumatosis intestinalis, mild intestinal dilation, mild intestinal ileus, thickened bowel walls, paucity/absence of bowel gas	mild to moderate abdominal distention	identical to above clinical finding criteria for NEC diagnosis	thrombocytopenia, leukocytosis or leukopenia, neutropenia, bandemia, heme-positive stool, metabolic acidosis

© 2019 Heath M et al. *JAMA Network Open.*

eTable 2. Study Criteria Used for Focal or Spontaneous Intestinal Perforation

Ranked order of study criteria met was (1) radiological signs, (2) abdominal signs, (3) clinical findings, and (4) lab findings.

STUDY CLASSIFICATION	RADIOLOGIC SIGNS	ABDOMINAL SIGNS	CLINICAL FINDINGS	LABORATORY FINDINGS
**Focal or spontaneous intestinal perforation**	intestinal perforation, pneumoperitoneum, no pneumatosis intestinalis	abdominal distention, abdominal wall discoloration	asymptomatic, hypotension	leukocytosis

© 2019 Heath M et al. *JAMA Network Open.*

eTable 3. Study Criteria Used to Define Pathogenic Infection Outside the Gastrointestinal Tract

Study classifications follow infection definitions from the US Center for Disease Control/National Healthcare Safety Network (CDC/NHSN). Ranked order of study criteria met was (1) laboratory findings and (2) clinical findings. Four types of infection are excluded and not reported in this study: (i) early onset sepsis (before 72 hrs age), as antibiotics were prophylactically administered to infants at birth in all three hospital centers; (ii) a bloodstream infection that is identified secondary to another site of infection; (iii) infections associated with use of a central line; and (iv) laboratory detection of the genera *Blastomyces*, *Histoplasma*, *Coccidioides*, *Paracoccidioides*, *Cryptococcus*, and *Pneumocystis*, which typically give rise to community-associated infections and are rarely known to cause healthcare-associated infections.

STUDY CLASSIFICATION	LABORATORY FINDINGS	CLINICAL FINDINGS
**NEONATAL SEPSIS, LATE-ONSET**	After seventy-two hours of age:<br>bacterial, viral, or fungal pathogen, which is not included on the NHSN common commensal list, confirmed in 1 or more blood or serum specimens<br>confirmation obtained by culture or non-culture microbiological testing methods<br>organism identified is not related to an infection at another site	nonspecific, including but not limited to:<br>temperature irregularity,<br>respiratory distress,<br>hypotension,<br>feeding intolerance,<br>glucose intolerance,<br>neurological changes (lethargy/irritability, seizures, hypotonia),<br>abnormal perfusion,<br>bleeding issues,<br>unexplained jaundice
**OTHER CONFIRMED NON-GI INFECTIONS**	confirmed bacterial, viral, or fungal pathogen in sterile body fluids, such as cerebrospinal fluid (CSF), tracheal aspirate, urine, and/or joint fluid	identical to above clinical finding criteria for sepsis
**INFECTION NEGATIVE**		
suspected infection	leukocytosis or leukopenia,<br>elevated immature neutrophil counts,<br>low absolute neutrophil count,<br>thrombocytopenia,<br>serial elevated C-reactive protein<br>serum alkaline phosphatase	identical to above clinical finding criteria for sepsis
no concern regarding infection	no laboratory tests were ordered	asymptomatic

© 2019 Heath M et al. *JAMA Network Open.*

eTable 4. Summary of NEC Cohorts at Different Clinical Sites

The number of enrolled infants who were clinically designated as NEC suspicion or severe NEC are provided per hospital site. Cohort values shown are the total number of subjects enrolled at each site, grouped by gestational age (GA) bin and the number of cases requiring surgical intervention (peritoneal drain or laparotomy). Percentages, in italics and within parentheses, are related to the number of cases divided by all subjects by cohort and gestational age at birth. Number of NEC cases and suspicion was stratified by either being <27 weeks or ≥27 weeks GA at birth. Median and accompanying interquartile values, in italics and parentheses, are used to identify the post-conceptual age and day of age for cohorts and gestational age at birth. Values and relative percentages are provided for the number of mortalities associated with necrotizing enterocolitis. Abbreviations: surg inter, surgical intervention; CH, Children's Hospital of New Orleans, LA; TI, Touro Infirmary, New Orleans, LA; WU, Washington University in St. Louis, St. Louis, Missouri.

**COHORT**				**SUSPECTED NEC**				**SEVERE NEC**			
					**at time of initial episode**				**at time of initial episode**		
**site**	**GA** (wks)	**# infants at risk**	**# surg inter**	**# infants**	**PCA** (wks)	**day of age**	**weight** (g)	**# infants**	**PCA** (wks)	**day of age**	**weight** (g)
CH	< 27	14*	2 *(14%)*	1 *(7%)*	29. 3	37	901	2 *(14%)*	32.6 *(31.8-33.4)*	49 *(47-50)*	1463 *(1274-1651)*
	≥ 27	15	2 *(13%)*	5 *(33%)*	33.6 *(30.9-35.4)*	27 *(23-29)*	1464 *(1130-1600)*	3 *(20%)*	33.1 *(32.1-33.7)*	31 *(19-32)*	1620 *(1390-1705)*
	total	29	3 *(14%)*	6 *(21%)*	32.2 *(29.7-35.0)*	28 *(24-35)*	1297 *(958-1566)*	5 *(17%)*	33.1 *(31.0-34.1)*	33 *(31-45)*	1620 *(1159-1790)*
TI	< 27	22	0	1 *(4%)*	26.9	13	553	2 *(9%)*	34.7	66	1913
	≥ 27	46	3 *(7%)*	9 *(20%)*	29.9 *(29-30.7)*	9 *(5-12)*	1043 *(994-1105)*	4 *(9%)*	32.4 *(30.7-34.6)*	13 *(9-16)*	1310 *(1165-1480)*
	total	68	3 *(4%)*	*10* *(15%)*	29.9 *(28.6-30.7)*	9 *(5-12)*	1043 *(894-1104)*	6 *(9%)*	33.8 *(31.7-35.0)*	17 *(11-49)*	1516 *(1250-1623)*
WU	< 27	17	4 *(24%)*	3 *(18%)*	26.9 *(26.9-27.9)*	19 *(16-20)*	820 *(765-860)*	8 *(47%)*	27.1 *(26.9-34.0)*	21 *(16-59)*	1075 *(704-1491)*
	≥ 27	22**	0	0	-	-	-	6 *(27%)*	37.7 *(35.6-40.1)*	17 *(9-41)*	2310 *(2093-2565)*
	total	39	4 *(10%)*	3 *(8%)*	26.9 *(26.9-27.9)*	19 *(16-20)*	820 *(765-860)*	14 *(36%)*	35 *(27.0-37.5)*	21 *(10-46)*	1705 *(1040-2255)*
All	< 27	53	6 *(11%)*	5 *(9%)*	26.9 *(26.9-28.4)*	19 *(13-21)*	820 *(710-880)*	12 *(22%)*	*32.4* *(27.1-34.3)*	49 *(20-62)*	1265 *(960-1739)*
	≥ 27	83	5 *(6%)*	14 *(17%)*	30.7 *(29.1-32.9)*	12 *(6-26)*	1103 *(999-1421)*	13 *(16%)*	34.3 *(33.1-37.0)*	16 *(9-31)*	1650 *(1430-2220)*
	total	136	11 *(8%)*	19 *(14%)*	29.4 *(28.6-30.9)*	13 *(8-25)*	1015 *(873-1254)*	25 *(18%)*	33.9 *(31.0-35.7)*	21 *(10-52)*	1620 *(1110-2050)*

* Records and samples for one enrollee were removed from the study after custodial transfer of infant to state care.

** Two infants discontinued participation in the study at PCA week 32 at parental request: data (disease, surgery, mortality, etc.) after PCA wk 32 were not included. Neither infant had NEC, sepsis, confirmed infection at non-GI site, or SIP.

© 2019 Heath M et al. *JAMA Network Open.*

eTable 5. List of 25 Radiologically Confirmed (Severe) Cases of Necrotizing Enterocolitis Enrolled

Criteria for defining severe NEC are shown in Table e1; primary criteria was confirmation by radiographic evidence. Abbreviations for parent race/ethnicity self-identification: B=African American/Black; W=Caucasian/White; and H=Hispanic. Other abbreviations are: GA=gestational age; M=male; F=female; C=Cesarean delivery; V=vaginal delivery

**subject ID**	**GA** (wks)	**birth weight** (g)	**sex**	**race / ethnicity**	**birth delivery**	**at time of initial NEC diagnosis**			**Bell stage**	**demise in NICU**
						**PCA** (wks)	**day of life**	**weight** (g)		
C1	29.9	700	F	B	C	34.3	31	1159	2 A	no
C2	24.6	650	F	B	C	31.0	45	1085	2 B	no
C3	26.9	991	M	B	V	34.1	52	1840	2 A	no
C4	28.4	1013	F	B	V	33.1	33	1790	2 B	no
C5	30.1	1620	M	B	V	31.0	7	1620	3 B	no
T1	30.0	1240	M	W	C	31.0	7	1190	2 B	no
T2	28.7	1010	F	H	C	29.9	9	1090	2 B	no
T3	32.4	1440	F	B	V	33.9	16	1430	3 B	no
T4	25.3	815	M	B	C	33.7	59	1602	2 A	no
T5*	25.0	855	M	B	C	35.7	73	2234	2 A	no
T6	34.6	1380	M	H	C	37.0	17	1630	3 B	no
W1	34.0	2360	F	W	C	35.1	9	2050	2 A	no
W2	24.3	700	F	B	C	25.1	7	720	2 B	no
W3	31.1	1190	F	W	C	34.3	24	1650	2 B	no
W4	26.0	480	M	W	C	33.7	55	1420	2 B	yes
W5	36.7	2565	M	W	V	43.1	46	2620	2 B	no
W6	36.3	2330	F	W	C	37.0	6	2220	2 B	no
W7	24.3	750	M	W	C	27.1	21	1110	3 B	no
W8	24.3	650	F	W	C	27.1	21	1040	3 A	yes
W9	24.7	640	M	W	C	27.1	18	630	2 B	no
W10*	24.6	630	F	W	C	25.9	10	655	3 A	yes
W11	27.6	790	M	W	C	40.7	93	2700	3 B	yes
W12* ≠	37.1	2530	F	W	C	38.4	10	2400	2 A	no
W13*	25.0	760	M	B	C	34.9	69	2255	2 A	no
W14*	23.6	430	M	W	C	37.6	99	1705	2 B	no

* No samples were obtained during NEC period. If a sample was not obtained during the radiologically- and clinically-defined period of NEC, all samples from the infant were excluded from cross-sectional analyses.

≠ Study subject was excluded due to gestational age >37 weeks at time of enrollment

© 2019 Heath M et al. *JAMA Network Open.*

eTable 6. List of 19 Suspected Necrotizing Enterocolitis Cases Enrolled

Criteria for defining suspected NEC are shown in Table e1; these cases could not be confirmed by radiographic evidence. Bell stage documented by attending neonatologist in medical record is shown and may differ from our manuscript criteria. Abbreviations for parent race/ethnicity self- identification: B=African American/Black; W=Caucasian/White; and H=Hispanic. Other abbreviations: GA=gestational age; M=male; F=female; C=Cesarean delivery; V=vaginal delivery

**subject ID**	**GA** (wk)	**birth weight** (g)	**sex**	**race / ethnicity**	**birth delivery**	**at time of clinical diagnosis of NEC suspicion**			**Bell stage**	**demise in NICU**
						**PCA** (wks)	**day of life**	**weight** (g)		
C6	28.0	810	M	B	V	28.9	8	885	1	no
C7	29.4	1340	M	B	V	33.6	29	1740	1	no
C8*	29.4	1130	F	W	C	35.4	42	1600	1	no
C9	24.0	715	M	B	V	29.3	37	901	1	no
C10	32.7	1170	F	B	C	36.0	23	1464	1	no
C11*	27.0	850	M	B	C	30.9	27	1130	1	no
T7*	30.0	1200	F	W	C	30.7	6	1105	1	no
T8	28.7	1120	M	B	C	29.4	5	1070	1	no
T9	29.1	1485	M	B	C	30.7	11	1435	1	no
T10	32.3	1420	M	B	C	35.0	2	1377	1	no
T11	29.6	1170	M	B	C	30.3	5	994	1	no
T12*	28.9	1190	F	W	V	30.6	12	1100	1	no
T13	25.0	560	F	B	V	26.9	13	553	1	no
T14	26.7	531	F	B	C	27.7	7	490	1	no
T15	26.7	860	M	B	C	28.4	12	860	1	no
T16	26.9	940	M	W	C	29.0	29	1015	1	no
W15**	24.3	700	M	W	C	26.9	19	900	3 B	yes
W16	26.0	830	F	B	C	28.9	21	710	1	no
W17	25.1	790	M	B	C	26.9	13	820	1	no

* No samples were obtained during NEC period. If a sample was not obtained during the radiologically- and clinically-defined period of NEC, all samples from the infant were excluded from cross-sectional analyses.

** No abdominal radiographic evidence nor abdominal ultrasound evidence of pneumatosis intestinalis, portal venous gas, or pneumoperitoneum on multiple evaluations.

© 2019 Heath M et al. *JAMA Network Open.*

eTable 7. List of 3 Enrolled Infants With Spontaneous Intestinal Perforation (SIP) and Necrotizing Enterocolitis

Abbreviations for parent race/ethnicity self-identification: B=African American/Black; W=Caucasian/White; and H=Hispanic. Other abbreviations: GA=gestational age at birth; M=male; F=female; C=Cesarean delivery; V=vaginal delivery; and NA=not applicable

subject ID	GA (wk)	birth weight (g)	sex	race	at time of SIP diagnosis			At time of NEC diagnosis or suspicion					
					PCA (wks)	day of life	weight (g)	# days after SIP	PCA (wks)	day of life	weight (g)	study classification	Bell stage
C1	29.9	700	F	B	30.4	5	800	26	34.3	31	1159	diagnosis	2A
C7	29.4	1340	M	B	30.4	7	1360	22	33.6	29	1740	suspicion	1
W11	27.6	790	M	W	28.4	7	940	86	40.7	93	2700	diagnosis	3B

© 2019 Heath M et al. *JAMA Network Open.*

eTable 8. List of 86 Enrolled Infants Who Were Neither Clinically Diagnosed With nor Suspected of Having Necrotizing Enterocolitis

Abbreviations for parent race/ethnicity self-identification: B=African American/Black; W=Caucasian/White; and H=Hispanic. Other abbreviations: GA=gestational age; M=male; F=female; C=Cesarean delivery; V=vaginal delivery

**subject ID**	**GA** (wks)	**birth weight** (g)	**sex**	**race / ethnicity**	**birth delivery**	**demise in NICU**
C13	25.0	710	F	B	C	no
C14	24.4	710	F	B	C	no
C15	24.6	715	M	B	C	no
C16	27.1	915	F	B	V	no
C17	23.9	490	F	B	V	no
C18	26.0	855	M	B	C	no
C19	27.0	950	F	H	C	no
C20	31.7	1289	M	W	C	no
C21	30.9	1390	M	B	C	no
C22	24.1	630	M	W	C	no
C23	27.0	1075	F	H	C	no
C24	30.0	1489	M	B	V	no
C25	26.0	589	F	B	C	no
C26	31.6	1435	F	W	C	no
T17	24.6	655	M	B	V	no
T18	25.9	845	M	B	V	no
T19	25.1	680	M	W	V	no
T20	29.7	1035	M	B	C	no
T21	29.9	1075	F	B	C	no
T22	30.0	790	F	W	C	no
T23	25.3	540	F	B	C	no
T24	23.6	540	M	B	C	no
T25	27.4	1500	F	B	V	no
T26	28.7	1100	M	B	C	no
T27	26.9	800	F	W	C	no
T28	25.6	805	F	B	C	no
T29	24.6	655	M	W	V	no
T30	26.9	1025	F	B	C	yes
T31	35.3	1240	F	B	C	no
T32	30.0	850	M	H	C	no
T33	30.0	1330	M	B	C	no
T34	34.1	2450	F	B	C	no
T35	34.1	2520	F	B	C	no
T36	30.9	1390	M	B	C	no
T37	26.4	910	F	B	V	no
T38	26.0	915	F	B	C	no

© 2019 Heath M et al. *JAMA Network Open.*

eTable 8 *(continued)*. List of 86 Enrolled Infants Who Were Neither Clinically Diagnosed With nor Suspected of Having Necrotizing Enterocolitis

Abbreviations for parent race/ethnicity self-identification: B=African American/Black; W=Caucasian/White; and H=Hispanic. Other abbreviations: GA=gestational age; M=male; F=female; C=Cesarean delivery; V=vaginal delivery

subject ID	GA (wks)	birth weight (g)	sex	race / ethnicity	birth delivery	demise in NICU
T39	25.0	625	M	B	C	no
T40	26.9	1000	M	W	C	no
T41	26.4	985	M	B	V	no
T42	37.0	2703	F	B	V	no
T43	28.1	1050	M	B	C	no
T44	29.1	1310	F	B	C	no
T45	29.1	1265	M	B	C	no
T46	29.7	1350	M	B	V	no
T47	26.4	845	F	B	C	no
T48	28.4	1135	M	B	C	no
T49	29.6	1291	M	W	C	no
T50	29.3	1270	M	B	C	no
T51	30.3	1150	M	B	C	no
T52	32.1	1450	M	B	V	no
T53	30.3	1020	F	B	C	no
T54	32.4	1050	F	B	V	no
T55	32.6	1525	F	B	C	no
T56	32.6	1490	F	B	C	no
T57	32.4	1220	F	B	C	no
T58	32.3	1140	F	B	C	no
T59	31.1	1335	F	B	C	no
T60	32.4	1160	F	B	C	no
T61	32.3	1770	M	W	C	no
T62	34.1	1470	F	B	C	no
T63	34.3	1230	M	B	C	no
T64	35.6	1210	M	B	C	no
T65	37.1	2320	M	B	C	no
T66	32.3	1380	M	B	C	no
W18	26.7	930	F	W	C	yes
W19	29.9	1700	F	W	V	no
W20	25.0	670	F	B	C	no
W21	26.4	790	F	B	V	no
W22	29.4	1150	F	W	C	no
W23	29.3	1400	M	W	V	no
W24	27.3	1160	M	W	C	no

© 2019 Heath M et al. *JAMA Network Open.*

eTable 8 *(continued)***.** List of 86 Enrolled Infants Who Were Neither Clinically Diagnosed With nor Suspected of Having Necrotizing Enterocolitis

Abbreviations for parent race/ethnicity self-identification: B=African American/Black; W=Caucasian/White; and H=Hispanic. Other abbreviations: GA=gestational age; M=male; F=female; C=Cesarean delivery; V=vaginal delivery

**subject ID**	**GA** (wks)	**birth weight** (g)	**sex**	**race / ethnicity**	**birth delivery**	**demise in NICU**
W25	27.3	1060	M	W	C	no
W26	29.3	1580	F	W	C	no
W27	31.9	2200	M	W	V	no
W28	27.4	1110	F	W	V	no
W29	31.9	1565	F	B	V	no
W30	26.0	1030	F	B	V	no
W31	29.1	1400	M	W	V	no
W32	29.1	1370	F	W	V	no
W33	26.6	890	F	B	V	no
W34	29.6	1515	F	W	V	no
W35	28.1	1200	M	W	C	no
W36	27.9	1050	M	B	V	no
W37	27.9	1140	F	B	V	no
W38	25.4	630	F	B	C	no
W39	36.4	3161	M	W	C	no

© 2019 Heath M et al. *JAMA Network Open.*

eTable 9. Summary of Sepsis and Other Non–GI Tract Infection Cohorts at Different Clinical Sites

The number of enrolled infants who were clinically designated as having sepsis or other non-GI infection are provided per hospital site.[3,4] Cohort values shown are the total number of subjects enrolled at each site, grouped by gestational age (GA) bin. Percentages, in italics and within parentheses, are related to the number of cases divided by all subjects by cohort and gestational age at birth. Median and accompanying interquartile values, in italics and parentheses, are used to identify the post-conceptual age, day of age, and weight at time of infection. Abbreviations: GA=gestational age at birth; CH, Children's Hospital of New Orleans, LA; TI, Touro Infirmary, New Orleans, LA; WU, Washington University in St. Louis, St. Louis, Missouri.

COHORT			OTHER NON-GI INFECTIONS				SEPSIS			
site	GA (wks)	# infants at risk	# infants	episode closest to NEC			# infants	episode closest to NEC		
				PCA (wks)	day of age	weight (g)		PCA (wks)	day of age	weight (g)
CH	< 27	14	4 *(21%)*	28.4 *(26.9-30.4)*	17 *(12-38)*	900 *(780-1130)*	4 *(29%)*	31.6 *(27.8-35.9)*	46 *(13-80)*	1375 *(978-1725)*
	≥ 27	15	4 *(27%)*	31.6 *(30.9-32)*	32 *(25-34)*	1154 *(1061-1198)*	0	0	0	0
	total	29	8 *(28%)*	31.6 *(28.6-32)*	31 *(12-37)*	1030 *(878-1219)*	4 *(14%)*	31.6 *(27.8-35.9)*	46 *(13-80)*	1375 *(978-1725)*
TI	< 27	22	5 *(23%)*	29 *(28.6-29.6)*	26 *(26-28)*	960 *(955-1030)*	6 *(27%)*	28.3 *(27.3-28.9)*	16 *(9-27)*	820 *(623-926)*
	≥ 27	46	2 *(4%)*	32.2	29	2045	6 *(13%)*	32.3 *(31.8-33)*	17 *(13-22)*	1303 *(1200-1407)*
	total	68	7 *(10%)*	29.6 *(28.8-32.2)*	26 *(25-33)*	1030 *(958-1565)*	12 *(18%)*	30.6 *(28.4-32.1)*	17 *(10-24)*	1140 *(830-1266)*
WU	< 27	17	1 *(6%)*	25.3	8	720	8 *(47%)*	*30* *(28.6-33.6)*	41 *(24-56)*	1070 *(958-1310)*
	≥ 27	22	0	0	0	0	2 *(9%)*	*35.6* *(34.8-36.3)*	19 *(13-24)*	2150 *(2115-2185)*
	total	39	1 *(3%)*	25.3	8	720	10 *(26%)*	*32* *(28.7-35.4)*	32 *(22-51)*	*1108* *(980-2034)*
All	< 27	53	7 *(13%)*	28.4 *(26.4-28.8)*	23 *(13-26)*	900 *(708-958)*	18 *(34%)*	28.8 *(28-32)*	27 *(15-51)*	1000 *(810-1194)*
	≥ 27	83	6 *(7%)*	31.6 *(31.6-32.6)*	32 *(23-37)*	1219 *(1142-1575)*	8 *(10%)*	33 *(31.8-34.8)*	17 *(12-24)*	1397 *(1220-2115)*
	total	136	15 *(11%)*	29 *(28.4-31.7)*	26 *(17-32)*	960 *(855-1260)*	26 *(19%)*	31.1 *(28.5-33.8)*	22 *(13-45)*	1108 *(951-1630)*

© 2019 Heath M et al. *JAMA Network Open.*

eTable 10. List of All 26 Late-Onset Neonatal Sepsis Cases Enrolled

Sepsis event was either closest in time to NEC episode or after study enrollment. Abbreviations: GA=gestational age at birth; M=male; F=female; B=African American/Black, W=Caucasian/White, H=Hispanic, C=Cesarean delivery; V=vaginal delivery; NA=not applicable.

**subject ID**	**GA** (wk)	**birth weight** (g)	**sex**	**race / ethnicity**	**birth delivery**	**at time of sepsis**		**total no. days antibiotics received** (% of NICU stay)	**NEC Bell stage** (if applicable)	**demise in NICU**
						**PCA** (wks)	**day of life**			
C3*	26.9	991	M	B	V	28	8	19 *(25%)*	2 A	no
C13	25.0	710	F	B	C	27.1	15	28 *(30%)*	NA	no
C14	24.4	710	F	B	C	35.3	76	31 *(20%)*	NA	no
C15	24.6	715	M	B	C	37.6	91	61 *(33%)*	NA	no
T2	28.7	1010	F	H	C	29.9	9	23 *(12%)*	2 B	no
T4	25.3	815	M	B	C	28.4	22	20 *(23%)*	2 A	no
T6	34.6	1380	M	H	C	41.1	46	44 *(17%)*	3 B	no
T7	30.0	1200	F	W	C	31.9	13	30 *(5%)*	1	no
T13	25.0	560	F	B	V	29.0	28	19 *(22%)*	1	no
T14	26.7	531	F	B	C	28.1	10	32 *(39%)*	1	no
T17	24.6	655	M	B	V	25.6	7	30 *(16%)*	NA	no
T18	25.9	845	M	B	V	27.0	8	18 *(19%)*	NA	no
T19*	25.1	680	M	W/B	V	31.3	43	47 *(29%)*	NA	no
T20	29.7	1305	M	B	C	32.7	21	6 *(12%)*	NA	no
T21	29.9	1075	F	B	C	31.7	13	10 *(27%)*	NA	no
T22	30.0	790	F	W	C	33.1	22	17 *(30%)*	NA	no
W2*	24.3	700	F	B	C	29.1	34	20 *(20%)*	2 B	no
W4*	26.0	480	M	W	C	32.9	48	36 *(46%)*	2 B	yes
W6	36.3	2330	F	W	C	37.1	7	8 *(57%)*	2 B	no
W8	24.3	650	F	W	C	27.1	21	10 *(48%)*	3 A	yes
W14*	23.6	430	M	W	C	31.0	52	49 *(31%)*	2 B	no
W17*	25.1	790	M	B	C	35.9	75	14 *(14%)*	1 B	no
W18*	26.7	930	F	W	C	36.0	66	17 *(23%)*	NA	yes
W19*	29.9	1700	F	W	V	34.0	30	10 *(20%)*	NA	no
W20*	25.0	670	F	B	C	25.0	1	25 *(19%)*	NA	no
W21*	26.4	790	F	B	V	28.6	16	31 *(22%)*	NA	no

* * No samples were obtained during sepsis period. If a sample was not obtained during disease, all samples from the infant were excluded only from cross-sectional analyses of sepsis.

© 2019 Heath M et al. *JAMA Network Open.*

eTable 11. List of All 14 Cases of Confirmed, Non–GI Tract Infections in Urine, Bone, or Trachea

Abbreviations: GA=gestational age at birth; PCA=post-conceptual age; M=male; F=female; C=Cesarean delivery; V=vaginal delivery; T=trachea; B=bone; U=urine; S=skin.

**subject ID**	**GA** (wk)	**birth weight** (g)	**sex**	**race / ethnicity**	**birth delivery**	**at time of infection**		**total no. days antibiotics received** (% of NICU stay)	**NEC Bell stage** (if applicable)	**site of infection**
						**PCA** (wks)	**day of life**			
C6	28.0	810	M	B	V	28.7	5	13 *(11%)*	1	T
C11	27.0	850	M	B	C	32.9	41	18 *(18%)*	1	T
C16	27.1	915	F	B	V	31.6	31	25 *(19%)*	1	B,T
C17	23.9	490	F	B	V	32.3	59	36 *(27%)*	NA	T
C18	26.0	855	M	B	C	28.4	17	30 *(19%)*	NA	T
C19	27.0	950	F	H	C	31.7	32	7 *(10%)*	NA	T
T5	25.0	855	M	B	C	28.6	23	13 *(15%)*	2 A	T
T23	25.3	540	F	B	C	29.0	26	15 *(18%)*	NA	T
T24*	23.6	540	M	B	C	27.3	26	30 *(18%)*	NA	T
T25*	27.4	1500	F	B	V	32.9	38	8 *(16%)*	NA	S
T26*	28.7	1100	M	B	C	31.6	20	18 *(21%)*	NA	T
T27	26.9	800	F	W	C	34.3	52	8 *(8%)*	NA	U
T28	25.6	805	F	B	C	29.6	28	10 *(13%)*	NA	S
W2*	24.3	700	F	B	C	25.3	8	20 *(10%)*	2 B	T

* No samples were obtained during non-GI infection period. If a sample was not obtained during infection, all samples from the infant were excluded only from cross-sectional analyses of sepsis.

© 2019 Heath M et al. *JAMA Network Open.*

eTable 12. Accuracy and Reproducibility of In Vitro Measurements of Gut Lumen Content

Reference standard for total protein concentration was bovine serum albumin; expected absorbance (ABS) calibration standard was determined using the extinction coefficient for bovine serum albumin (43,824 M^{-1} cm^{-1}) and Beer's law. Reference standard for biochemical activity was 4-methylumbelliferyl phosphate; expected relative fluorescence units were provided by kit manufacturer. Median experimental measurement, reproducibility, and accuracy for each analyte are shown. P-values ≥0.05 indicate there is no significant difference between measurements. Abbreviations: ABS=absorbance; RFU=relative fluorescence units; SE=standard error; SI=small intestine; ND=not determined

ASSAY	RAW DATA			REPRODUCIBILITY		ACCURACY	
**total protein concentration in stool supernatant** *(Bradford assay)*	reference standard (mg/ml)	expected $ABS_{595\ nm}$	median experimental $ABS_{595\ nm}$	inter-operator SE	p-value	% deviation from absolute value	p-value
	0.750	0.166	0.176	0.010	0.52	4	0.52
	0.500	0.111	0.123	0.007	0.43	1	0.81
	0.250	0.055	0.055	0.003	0.52	10	0.18
	0.125	0.027	0.220	0.002	0.72	28	0.0003
	0.025	0.004	0.002	0.001	0.10	70	<0.0001
	0.000	0.000	0.001	0.001	0.22	50	0.0002
**iAP biochemical activity** *(MUP assay)*	reference standard (nmol)	expected RFU	median experimental RFU	inter-operator SE	p-value	% deviation from absolute value	p-value
	0.5	20,957,775	19,915,897	1,685,719	0.28	5	0.55
	0.4	16,951,461	16,448,436	839,613	0.45	2	0.72
	0.3	12,945,147	12,900,112	757,993	0.27	1	0.90
	0.2	8,938,833	8,419,157	630,068	0.47	3	0.70
	0.1	4,932,519	4,555,401	298,513	0.34	0	0.99
	0	926,205	85,588	2,049	0.88	91	0.008
**iAP abundance** *(immunoblot)*	reference standard	expected concentration (µg)	median experimental RFU	inter-operator SE	p-value	% deviation from absolute value	p-value
	human SI lysate	3.75	207,055	31,862	0.72	ND	ND
	purified calf iAP	3.00	703	113	0.11	ND	ND

© 2019 Heath M et al. *JAMA Network Open.*

eTable 13. IAP Measurements From 20 Stool Samples at the Time of Severe Necrotizing Enterocolitis

subject ID	PCA at time of sample collection *(wk)*	weight at sample collection *(g)*	iAP activity *(μmol min^{-1} g^{-1} stool protein)*	relative iAP content *(% human SI)*
C1	34.3	1159	575	95.5
C2	31.0	1085	441	132.7
C3	34.3	1840	2195	30.8
C4	43.3	2825	70	162.8
C5	31.0	1620	217	50.0
T1	31.0	1190	222	103.4
T2	29.9	1090	648	10.2
T3	33.9	1430	135	71.0
T4	34.0	1641	478	216.3
T6	38.6	2580	54	213.2
W1	35.1	2050	23	242.2
W2	26.1	900	48	153.8
W3	34.3	1650	127	48.7
W4	34.6	1300	517	68.7
W5	43.9	3040	18	79.0
W6	37.1	2220	29	196.1
W7	27.7	1330	63	144.6
W8	27.1	1040	519	32.9
W9	39.6	2400	284	54.0
W11	40.9	2700	148	328.0

© 2019 Heath M et al. *JAMA Network Open.*

eTable 14. IAP Measurements From 15 Stool Samples at the Time of Necrotizing Enterocolitis Suspicion

**subject ID**	**PCA at time of sample collection** *(wk)*	**weight at sample collection** *(g)*	**iAP activity** *(μmol min^{-1} g^{-1} stool protein)*	**relative iAP content** *(% human SI)*
C6	29.1	855	6	317.8
C7	33.6	1740	227	129.5
C9	29.6	920	272	39.5
C10	36.0	1464	172	123.0
T8	29.4	1070	424	30.9
T9	30.7	1435	2075	366.9
T10	35.1	1378	520	212.9
T11	30.3	970	127	223.8
T13	26.9	553	460	28.3
T14	28.1	495	355	85.5
T15	28.7	870	608	158.3
T16	29.1	1005	970	14.7
W15	29.1	1305	2	584.5
W16	30.4	860	2307	117.7
W17	26.9	820	268	19.8

© 2019 Heath M et al. *JAMA Network Open.*

eTable 15. IAP Measurements From 86 Enrolled Infants Who Were Neither Clinically Diagnosed With nor Suspected of Having Necrotizing Enterocolitis

subject ID	PCA at time of sample collection *(wk)*	weight at sample collection *(g)*	iAP activity *(μmol min^{-1} g^{-1} stool protein)*	relative iAP content *(% human SI)*
C13	31.3	1580	1231	0.1
C14	39.0	2560	723	2.8
C15	40.1	2170	3195	10.7
C16	40.4	2950	1872	7.6
C17	35.1	1440	9752	0.9
C18	27.0	810	463	5.6
C19	34.1	1210	622	2.5
C20	33.6	1370	805	3.7
C21	28.9	780	786	7.4
C22	29.7	1090	784	4.4
C23	37.9	2725	1848	2.4
C24	30.7	1296	2695	9.5
C25	38.1	2270	7495	1.2
C26	39.3	2390	1243	0.7
T17	25.4	595	554	26.3
T18	26.9	840	470	27.9
T19	42.7	2660	6922	2.7
T20	30.4	1040	175	4.5
T21	30.7	1025	995	5.0
T22	31.9	900	1477	2.6
T23	26.7	600	405	5.9
T24	32.0	1705	2787	2.1
T25	31.3	1960	875	2.4
T26	37.3	1513	7393	4.6
T27	27.7	650	884	1.9
T28	27.0	790	344	0.3
T29	25.6	500	144	14.2
T30	28.0	990	386	4.2
T31	38.4	1660	2268	3.2
T32	30.0	850	83	2.6
T33	32.6	1727	669	3.4
T34	35.0	2285	1608	5.3
T35	34.7	2415	2303	6.3
T36	32.7	1476	61	3.8
T37	26.4	910	714	6.8
T38	26.7	750	354	11.0
T39	29.7	990	409	5.7

© 2019 Heath M et al. *JAMA Network Open.*

eTable 15 *(continued)*. IAP Measurements From 86 Enrolled Infants Who Were Neither Clinically Diagnosed With nor Suspected of Having Necrotizing Enterocolitis

**subject ID**	**PCA at time of sample collection** *(wk)*	**weight at sample collection** *(g)*	**iAP activity** *(μmol min^{-1} g^{-1} stool protein)*	**relative iAP content** *(% human SI)*
T40	31.3	1450	1219	26.5
T41	34.0	2160	723	0.9
T42	37.0	2703	170	4.6
T43	29.0	965	63	11.6
T44	29.6	1165	305	6.2
T45	34.0	2084	2244	6.5
T46	32.0	1450	350	1.0
T47	28.0	850	148	51.1
T48	35.9	2428	6904	0.8
T49	31.3	1270	603	3.2
T50	30.7	1215	660	11.0
T51	31.7	1180	180	2.9
T52	35.0	1790	337	2.1
T53	32.4	1122	117	2.5
T54	34.0	1114	1461	4.1
T55	38.6	2269	291	3.5
T56	33.9	1478	1898	1.5
T57	35.9	1809	2618	1.1
T58	33.1	1230	6310	15.6
T59	36.0	1837	80	7.9
T60	34.1	1299	654	2.7
T61	36.4	2357	383	1.0
T62	35.3	2040	7386	4.6
T63	35.4	1277	136	5.4
T64	37.9	1423	211	1.0
T65	37.7	2836	544	17.2
T66	34.9	1550	1527	22.6
W18	29.9	1240	40	4.9
W19	31.6	1600	132	17.8
W20	28.3	960	176	12.7
W21	30.0	1090	919	7.5
W22	33.4	1680	39	49.1
W23	31.4	1520	97	6.1
W24	31.0	1560	714	140.6

© 2019 Heath M et al. *JAMA Network Open.*

eTable 15 *(continued)***.** IAP Measurements From 86 Enrolled Infants Who Were Neither Clinically Diagnosed With nor Suspected of Having Necrotizing Enterocolitis

**subject ID**	**PCA at time of sample collection** *(wk)*	**weight at sample collection** *(g)*	**iAP activity** *(μmol min^{-1} g^{-1} stool protein)*	**relative iAP content** *(% human SI)*
W25	31.0	1460	357	46.3
W26	35.6	2550	656	5.1
W27	31.4	2040	214	3.2
W28	29.0	970	695	15.7
W29	33.4	1670	239	5.2
W30	26.6	960	376	12.7
W31	33.7	2070	76	6.0
W32	33.7	1924	172	6.6
W33	33.9	1875	40	7.4
W34	40.4	3720	2275	0.5
W35	28.6	1150	474	14.3
W36	30.7	1320	547	0.4
W37	28.7	1095	170	2.4
W38	26.1	565	205	209.0
W39	36.9	3260	916	0.3

© 2019 Heath M et al. *JAMA Network Open.*

eTable 16. Proteins Identified in Preterm Gut Lumen (N = 635)

Collected from non-NEC infant, who was 32.57 weeks postconceptual age and 1000 g weight, a stool sample was analyzed by shotgun mass spectrometry. Proteins are ordered by sumPEP score. Shown are descriptions and Uniprot ID of proteins with MS sumPEP scores of 1400 to 96.

UniProt ID	Description
Q9Y6R7	IgGFc-binding protein
P02768	serum albumin
P02787	serotransferrin
P01024	complement C3
P00450	ceruloplasmin
P14410	sucrase-isomaltase, intestinal
P98088	mucin-5AC
P01009	alpha-1-antitrypsin
P15144	aminopeptidase N
Q9HC84	mucin-5B
P02751	fibronectin
P01011	alpha-1-antichymotrypsin
P01008	antithrombin-III
P0DOX5	immunoglobulin gamma-1 heavy chain
A8K7I4	calcium-activated Cl channel regulator 1
P12821	angiotensin-converting enzyme
P02788	lactotransferrin
Q9UGM3	deleted in malignant brain tumors 1 protein
Q02817	mucin-2
Q16819	meprin A subunit alpha
P01859	immunoglobulin heavy constant gamma 2
P01023	alpha-2-macroglobulin
P25311	zinc-alpha-2-glycoprotein
P00747	plasminogen
Q6UWV6	ectonucleotide pyrophosphatase-phosphodiesterase family member 7
P09923	**intestinal-type alkaline phosphatase**
O43895	Xaa-Pro aminopeptidase 2
P0DOX7	immunoglobulin kappa light chain
P27487	dipeptidyl peptidase 4
P08473	neprilysin [OS=Homo sapiens]
P04264	keratin, type II cytoskeletal 1
Q8TDL5	BPI fold-containing family B member 1
P01834	immunoglobulin kappa constant
P01860	immunoglobulin heavy constant gamma 3
P55259	pancreatic secretory granule membrane major glycoprotein GP2
P02760	protein AMBP
P07911	uromodulin
P13646	keratin, type I cytoskeletal 13
P02766	transthyretin
P35237	serpin B6
Q08380	galectin-3-binding protein
P0C0L4	complement C4-A
P60174	triosephosphate isomerase
P47989	xanthine dehydrogenase/oxidase
P02538	keratin, type II cytoskeletal 6A
P19835	bile salt-activated lipase
P48668	keratin, type II cytoskeletal 6C
Q9BYF1	angiotensin-converting enzyme 2
P28838	cytosol aminopeptidase
P30740	leukocyte elastase inhibitor
P13645	keratin, type I cytoskeletal 10
P00738	haptoglobin
P04259	keratin, type II cytoskeletal 6B
P02763	alpha-1-acid glycoprotein 1
P01861	immunoglobulin heavy constant gamma 4
A8K2U0	alpha-2-macroglobulin-like protein 1
P29508	serpin B3
P02790	hemopexin
P35527	keratin, type I cytoskeletal 9
P06702	protein S100-A9
P01833	polymeric immunoglobulin receptor
P15085	carboxypeptidase A1
Q9H3R2	mucin-13
Q9BYE9	cadherin-related family member 2
P05543	thyroxine-binding globulin
P20742	pregnancy zone protein
P04217	alpha-1B-glycoprotein
Q13228	selenium-binding protein 1
P09848	lactase-phlorizin hydrolase
P01031	complement C5
P01876	immunoglobulin heavy constant alpha 1
P08779	keratin, type I cytoskeletal 16
Q6UX06	olfactomedin-4
P27216	annexin A13
P02533	keratin, type I cytoskeletal 14
P16444	dipeptidase 1
P09525	annexin A4
P02771	alpha-fetoprotein
O75882	attractin
P19652	alpha-1-acid glycoprotein 2
Q04609	glutamate carboxypeptidase 2
P08697	alpha-2-antiplasmin
P51884	lumican
P15086	carboxypeptidase B
P69892	hemoglobin subunit gamma-2
P29622	kallistatin
P06748	nucleophosmin
P13647	keratin, type II cytoskeletal 5

eTable 16 *(continued)* Proteins Identified in Preterm Gut Lumen (N = 635)

Shown are descriptions and Uniprot ID of proteins with MS sumPEP scores of 95.0 to 47.9.

UniProt ID	Description
P05155	plasma protease C1 inhibitor
P02753	retinol-binding protein 4
P06733	alpha-enolase
P0DOX8	immunoglobulin lambda-1 light chain
P0DOX2	immunoglobulin alpha-2 heavy chain
P69891	hemoglobin subunit gamma-1
P0DOY2	immunoglobulin lambda constant 2
P07148	fatty acid-binding protein, liver
Q9BXP8	pappalysin-2
P04406	glyceraldehyde-3-phosphate dehydrogenase
P15586	N-acetylglucosamine-6-sulfatase
P12830	cadherin-1
Q12864	cadherin-17
P05120	plasminogen activator inhibitor 2
P62158	calmodulin
P19440	glutathione hydrolase 1 proenzyme
P08185	corticosteroid-binding globulin
P17931	galectin-3
P19013	keratin, type II cytoskeletal 4
P60709	actin, cytoplasmic 1
P07858	cathepsin B
P19801	amiloride-sensitive amine oxidase
P49747	cartilage oligomeric matrix protein
P07477	trypsin-1
P07478	trypsin-2
P35908	keratin, type II cytoskeletal 2 epidermal
O95497	pantetheinase
O14983	sarcoplasmic/ER calcium ATPase 1
P05090	apolipoprotein D
P07998	ribonuclease pancreatic
P07686	beta-hexosaminidase subunit beta
P54802	alpha-N-acetylglucosaminidase
P41222	prostaglandin-H2 D-isomerase
Q15493	regucalcin
P02749	beta-2-glycoprotein 1
P02748	complement component C9
P07093	glia-derived nexin
P07996	thrombospondin-1
Q9Y2T3	guanine deaminase
P48052	carboxypeptidase A2
P05787	keratin, type II cytoskeletal 8
P05109	protein S100-A8
Q12805	EGF-containing fibulin-like extracellular matrix protein 1
P04118	colipase

UniProt ID	Description
Q2M2H8	probable maltase-glucoamylase 2
P04083	annexin A1
P62736	actin, aortic smooth muscle
P02675	fibrinogen beta chain
P08582	melanotransferrin
P10643	complement component C7
P62979	ubiquitin-40S ribosomal protein S27a
Q13867	bleomycin hydrolase
P15941	mucin-1
P09622	dihydrolipoyl dehydrogenase, mitochondrial
Q9UBG3	cornulin
Q8NFJ5	retinoic acid-induced protein 3
P09093	chymotrypsin-like elastase 3A
Q9NQ84	G-protein coupled receptor family C group 5 member C
Q07075	glutamyl aminopeptidase
Q07654	trefoil factor 3
Q07837	neutral and basic amino acid transport protein rBAT
Q03154	aminoacylase-1
Q10588	ADP-ribosyl cyclase/cyclic ADP-ribose hydrolase 2
Q9Y646	carboxypeptidase Q
P01042	kininogen-1
P04179	superoxide dismutase [Mn], mitochondrial
P13727	bone marrow proteoglycan
Q6UXC1	apical endosomal glycoprotein
P17538	chymotrypsinogen B
P04114	apolipoprotein B-100
P00390	glutathione reductase, mitochondrial
Q6FI13	histone H2A type 2-A
O75369	filamin-B
Q04695	keratin, type I cytoskeletal 17
P00338	L-lactate dehydrogenase A chain
O43451	maltase-glucoamylase, intestinal
O14818	proteasome subunit alpha type-7
P12955	Xaa-Pro dipeptidase
Q9ULA0	aspartyl aminopeptidase
Q99895	chymotrypsin-C
P50995	annexin A11
P04040	catalase
P04196	histidine-rich glycoprotein
P15311	ezrin
P35555	fibrillin-1
P07225	vitamin K-dependent protein S
Q12929	epidermal growth factor receptor kinase substrate 8
O43707	alpha-actinin-4

© 2019 Heath M et al. *JAMA Network Open.*

eTable 16 *(continued)*. Proteins Identified in Preterm Gut Lumen (N = 635)

Shown are descriptions and Uniprot ID of proteins with MS sumPEP scores of 47.6 to 13.0.

UniProt ID	Description
P05154	plasma serine protease inhibitor
Q9HBB8	cadherin-related family member 5
P13929	beta-enolase
P04908	histone H2A type 1-B/E
O43280	trehalase
A0A0C4DH31	immunoglobulin heavy variable 1-18
P02750	leucine-rich alpha-2-glycoprotein
Q14624	inter-alpha-trypsin inhibitor heavy chain H4
P07093	glia-derived nexin
P20933	N(4)-(beta-N-acetylglucosaminyl)-L-asparaginase
Q9H6S3	epidermal growth factor receptor kinase substrate 8-like protein 2
P08727	keratin, type I cytoskeletal 19
Q92820	gamma-glutamyl hydrolase
P23142	fibulin-1
P12532	creatine kinase U-type, mitochondrial
P61916	epididymal secretory protein E1
Q01546	keratin, type II cytoskeletal 2 oral
Q92485	acid sphingomyelinase-like phosphodiesterase 3B
P08861	chymotrypsin-like elastase family member 3B
P31151	protein S100-A7
Q03403	trefoil factor 2
P24855	deoxyribonuclease
P04004	vitronectin
Q96DA0	zymogen granule protein 16 homolog
P31025	lipocalin-1
Q9UGM5	fetuin-B
Q13822	ectonucleotide pyrophosphatase-phosphodiesterase family member 2
P08236	beta-glucuronidase
P02774	vitamin D-binding protein
P04278	sex hormone-binding globulin
Q9UHL4	dipeptidyl peptidase 2
P13866-1	sodium/glucose cotransporter 1
P00441	superoxide dismutase
P63104	14-3-3 protein zeta/delta
P80188	neutrophil gelatinase-associated lipocalin
Q9H3G5	probable serine carboxypeptidase

UniProt ID	Description
P40199	carcinoembryonic antigen-related cell adhesion molecule 6
Q08188	protein-glutamine gamma-glutamyltransferase E
P00751	complement factor B
P21589	5'-nucleotidase
P69905	hemoglobin subunit alpha
P27105	erythrocyte band 7 integral membrane protein
P01871	immunoglobulin heavy constant mu
P06744	glucose-6-phosphate isomerase
P28799	granulins
P00734	prothrombin
P16930	fumarylacetoacetase
P12429	annexin A3
P04054	phospholipase A2
Q99828	calcium and integrin-binding protein 1
Q9UQQ1	N-acetylated-alpha-linked acidic dipeptidase-like protein
P43652	afamin
Q14520	hyaluronan-binding protein 2
P07339	cathepsin D
P12273	prolactin-inducible protein
P02765	alpha-2-HS-glycoprotein
A0A0B4J1X5	immunoglobulin heavy variable 3-74
P16422	epithelial cell adhesion molecule
Q53GD3	choline transporter-like protein 4
P04432	immunoglobulin kappa variable 1D-39
O00391	sulfhydryl oxidase 1
Q96KP4	cytosolic non-specific dipeptidase
P08253	72 kDa type IV collagenase
A0A0C4DH29	immunoglobulin heavy variable 1-3
Q03591	complement factor H-related protein 1
P05556	integrin beta-1
Q9H0W9	ester hydrolase C11orf54
Q9NR71	neutral ceramidase
P40925	malate dehydrogenase, cytoplasmic
Q13162	peroxiredoxin
Q6EMK4	vasorin
P06731	carcinoembryonic antigen-related cell adhesion molecule 5

eTable 16 *(continued)*. Proteins Identified in Preterm Gut Lumen (N = 635)

Shown are descriptions and Uniprot ID of proteins with MS sumPEP scores of 30.8 to 21.3.

UniProt ID	Description
Q06830	peroxiredoxin-1
P06865	beta-hexosaminidase subunit alpha
P09211	glutathione S-transferase
P04745	alpha-amylase 1
Q695T7	sodium-dependent neutral amino acid transporter B(0)AT1
P02489	alpha-crystallin A chain
Q6UWP2	dehydrogenase/reductase SDR family member 11
P36980	complement factor H-related protein 2
P03973	antileukoproteinase
O60494	cubilin
Q6P1J6	phospholipase B1, membrane-associated
Q9HAT2	sialate O-acetylesterase
P01780	immunoglobulin heavy variable 3-7
P01824	immunoglobulin heavy variable 4-39
O14745	Na(+)/H(+) exchange regulatory cofactor NHE-RF1
P25789	proteasome subunit alpha type-4
P05546	heparin cofactor 2
P17900	ganglioside GM2 activator
P60900	proteasome subunit alpha type-6
Q5T2W1	Na/H exchange regulatory cofactor
P02679	fibrinogen gamma chain
P12882	myosin-1
P07358	complement component C8 β chain
P19827	inter-alpha-trypsin inhibitor heavy chain H1
A0A0B4J1Y9	immunoglobulin heavy variable 3-72
Q14508	WAP four-disulfide core domain protein 2
P01782	immunoglobulin kappa variable 3-9
P22352	glutathione peroxidase 3
O14638	ectonucleotide pyrophosphatase-phosphodiesterase family member 3
Q8N4F0	BPI fold-containing family B member 2
P13688	carcinoembryonic antigen-related cell adhesion molecule 1
Q99715	collagen alpha-1(XII) chain
Q8TE67	epidermal growth factor receptor kinase substrate 8-like protein 3
P12111	collagen alpha-3(VI) chain
P01019	angiotensinogen
P07195	L-lactate dehydrogenase B chain

UniProt ID	Description
Q8WUM4	programmed cell death 6-interacting protein
Q14315	filamin-C
P29377	protein S100-G
P04066	tissue alpha-L-fucosidase
P08174	complement decay-accelerating factor
P61626	lysozyme c
P36222	chitinase-3-like protein 1
Q86SQ4	adhesion G-protein coupled receptor
Q14002	carcinoembryonic antigen-related cell adhesion molecule 7
P09972	fructose-bisphosphate aldolase C
P01743	immunoglobulin heavy variable 1-46
Q01518	adenylyl cyclase-associated protein 1
P25788	proteasome subunit alpha type-3
O43490	prominin-1
P98160	basement membrane-specific heparan sulfate proteoglycan core protein
Q9NP55	BPI fold-containing family A member 1
P26038	moesin
P53990	IST1 homolog
P01614	immunoglobulin kappa variable 2D-40
P28066	proteasome subunit alpha type-5
P62937	peptidyl-prolyl cis-trans isomerase A
Q99497	protein/nucleic acid deglycase DJ-1
P11047	laminin subunit gamma-1
A0A0C4DH38	immunoglobulin heavy variable 5-51
P01624	immunoglobulin kappa variable 3-15
Q8TE67	epidermal growth factor receptor kinase substrate 8-like protein 3
P62258	14-3-3 protein epsilon
O00754	lysosomal alpha-mannosidase
P16233	pancreatic triacylglycerol lipase
A0A087WSY6	immunoglobulin kappa variable 3D-15
Q01459	di-N-acetylchitobiase
P01591	immunoglobulin J chain
A0A0C4DH73	immunoglobulin kappa variable 1-12
A0A0B4J1X8	immunoglobulin heavy variable 3-43
P20618	proteasome subunit beta type-1
P01764	immunoglobulin heavy variable 3-23

© 2019 Heath M et al. *JAMA Network Open.*

eTable 16 *(continued)*. Proteins Identified in Preterm Gut Lumen (N = 635)

Shown are descriptions and Uniprot ID of proteins with MS sumPEP scores of 21.2 to 13.1.

UniProt ID	Description
A0A0C4DH67	immunoglobulin kappa variable 1-8
A0A0B4J1V2	immunoglobulin heavy variable 2-26
P01619	immunoglobulin kappa variable 3-20
O75830	serpin I2
P61224	Ras-related protein Rap-1b
P06576	ATP synthase subunit beta
P01594	immunoglobulin kappa variable 1-33
P11678	eosinophil peroxidase
A0A0C4DH25	immunoglobulin kappa variable 3D-20
Q16651	prostasin
P62805	histone H4
O60635	tetraspanin-1
P29992	guanine nucleotide-binding protein subunit alpha-11
P78324	tyrosine-protein phosphatase non-receptor type substrate 1
P00915	carbonic anhydrase 1
P0DOX6	immunoglobulin mu heavy chain
Q15113	procollagen C-endopeptidase enhancer 1
Q9NZH0	G-protein coupled receptor family C group 5 member B
Q6YHK3	CD109 antigen
P01766	immunoglobulin heavy variable 3-13
A0A0A0MRZ8	immunoglobulin kappa variable 3D-11
P53634	dipeptidyl peptidase 1
A0A0B4J1V0	immunoglobulin heavy variable 3-15
P01040	cystatin-A
Q6W4X9	mucin-6
P23083	immunoglobulin heavy variable 1-2
Q15848	adiponectin
Q15274	nicotinate-nucleotide pyrophosphorylase
A0A0A0MS15	immunoglobulin heavy variable 3-49
P05156	complement factor I
A0A0J9YXX1	immunoglobulin heavy variable 5-10-1
P13671	complement component c6
P05121	plasminogen activator inhibitor 1
Q13219	pappalysin-1
P31947	14-3-3 protein sigma
Q9H4M9	EH domain-containing protein 1
P08294	extracellular superoxide dismutase
P01599	immunoglobulin kappa variable 1-17
P31949	protein S100-A11
P04155	trefoil factor 1
Q96A32	myosin regulatory light chain 2, skeletal muscle

UniProt ID	Description
P12814	alpha-actinin-1
P27482	calmodulin-like protein 3
Q13277	syntaxin-3
P61981	14-3-3 protein gamma
P09327	villin-1
P08962	CD63 antigen
O43653	prostate stem cell antigen
O00584	ribonuclease T2
A0A075B6S6	immunoglobulin kappa variable 2D-30
Q6UXY8	transmembrane channel-like protein 5
Q8WWA0	intelectin-1
Q9HD89	resistin
P45880	voltage-dependent anion-selective channel protein 2
P21796	voltage-dependent anion-selective channel protein 1
P17050	alpha-N-acetylgalactosaminidase
Q53R12	transmembrane 4
P11279	lysosome-associated membrane glycoprotein 1
Q86VB7	scavenger receptor cysteine-rich type 1 protein
Q9Y624	junctional adhesion molecule A
A0A075B6S2	immunoglobulin kappa variable 2D-29
Q14533	keratin, type II cuticular Hb1
P00505	aspartate aminotransferase
P01700	immunoglobulin lambda variable 1-47
P11142	heat shock cognate 71 kDa protein
P62070	Ras-related protein R-Ras2
P43251	biotinidase
P80748	immunoglobulin lambda variable 3-21
Q9Y5Y7	lymphatic vessel endothelial hyaluronic acid receptor 1
P06396	gelsolin
Q96NY7	chloride intracellular channel protein 6
P01034	cystatin-C
P12277	creatine kinase B-type
Q14393	growth arrest-specific protein 6
P01704	immunoglobulin lambda variable 2-14
P04632	calpain small subunit 1
P55287	cadherin-11
Q14CN2	Ca-activated chloride channel regulator
O75131	copine-3
P28070	proteasome subunit beta type-4
P25786	proteasome subunit alpha type-1
Q9UK41	vacuolar protein sorting-associated protein

eTable 16 *(continued)*. Proteins Identified in Preterm Gut Lumen (N = 635)

Shown are descriptions and Uniprot ID of proteins with MS sumPEP scores of 13.0 to 8.1.

UniProt ID	Description	UniProt ID	Description
P0C0S5	histone H2A.Z	P10619	lysosomal protective protein
P06312	immunoglobulin kappa variable 4-1	P01701	immunoglobulin lambda variable 1-51
O94760	N(G),N(G)-dimethylarginine dimethylaminohydrolase 1	Q92484	acid sphingomyelinase-like phosphodiesterase 3a
P36957	dihydrolipoyllysine-residue succinyltransferase	P22735	protein-glutamine gamma-glutamyltransferase K
A0A0B4J2H0	immunoglobulin heavy variable 1-69D	Q8NCR9	clarin-3
Q99102	mucin-4	P09466	glycodelin
P12235	ADP/ATP translocase 1	P37837	transaldolase
P25774	cathepsin S	P55058	phospholipid transfer protein
Q8WXI7	mucin-16	P08603	complement factor H
P31946	14-3-3 protein beta/alpha	P07360	complement component C8 γ chain
Q9UBC5	unconventional myosin-Ia	P14618	pyruvate kinase
Q9Y376	calcium-binding protein 39	P06732	creatine kinase M-type
P10909	clusterin	P60953	cell division control protein 42
Q6P4A8	phospholipase B-like 1	P30048	thioredoxin-dependent peroxide reductase, mitochondrial
Q9Y6N9	harmonin	P29401	transketolase
P61604	10 kDa HSP, mitochondrial	Q8IWL2	pulmonary surfactant-associated protein
P07357	complement component C8 α chain	O75367	core histone macro-H2A.1
Q9HCY8	protein S100-A14	Q969X1	protein lifeguard 3
Q92824	proprotein convertase subtilisin	O15551	claudin-3
P62873	guanine nucleotide-binding protein G(I)/G(S)/G(T) subunit beta-1	Q92484	acid sphingomyelinase-like phosphodiesterase 3a
Q14CN4	keratin, type II cytoskeletal 72	Q16891	MICOS complex subunit Mic60
P09960	leukotriene A-4 hydrolase	P09668	pro-cathepsin H
P51688	N-sulphoglucosamine sulphohydrolase	P43234	cathepsin O
P00813	adenosine deaminase	P00558	phosphoglycerate kinase 1
P78417	glutathione S-transferase omega-1	Q8TBG9	synaptoporin
Q5JS37	NHL repeat-containing protein 3	P56199	integrin alpha-1
P07602	prosaposin	P02452	collagen alpha-1(I) chain
Q01970	1-phosphatidylinositol 4,5-bisphosphate phosphodiesterase	P13284	gamma-interferon-inducible lysosomal thiol reductase
A0A0C4DH68	immunoglobulin kappa variable 2-24	P05783	keratin, type I cytoskeletal 18
Q01628	interferon-induced transmembrane protein 3	P43353	aldehyde dehydrogenase family 3 member B1
Q96JP2	unconventional myosin-XVB	Q6P5W5	zinc transporter ZIP4
P32119	peroxiredoxin-2	P07098-3	gastric triacylglycerol lipase
P16671	platelet glycoprotein 4	P13798	acylamino-acid-releasing enzyme
P21980	protein-glutamine gamma-glutamyltransferase 2	P08833	insulin-like growth factor-binding protein
P50395	Rab GDP dissociation inhibitor beta	P07237	protein disulfide-isomerase
A0A0A0MT36	immunoglobulin kappa variable 6D-21	P18206	vinculin
P23528	cofilin	P06870	kallikrein
A0A0C4DH24	immunoglobulin kappa variable 6-21	Q9H190	syntenin
Q14766	latent-transforming growth factor beta-binding protein 1	Q13126	S-methyl-5'-thioadenosine phosphorylase
P51148	Ras-related protein Rab-5C	P29972	aquaporin

© 2019 Heath M et al. *JAMA Network Open.*

eTable 16 *(continued)*. Proteins Identified in Preterm Gut Lumen (N = 635)

Shown are descriptions and Uniprot ID of proteins with MS sumPEP scores of 8.1 to 5.4.

UniProt ID	Description
P11234	Ras-related protein Ral-B
O75340	programmed cell death protein 6
P35247	pulmonary surfactant-associated protein D
Q14651	plastin-1
O15484	calpain-5
P05062	fructose-bisphosphate aldolase B
Q9H444	charged multivesicular body protein 4b
P01714	immunoglobulin lambda variable 3-19
Q2WGJ9	fer-1-like protein 6
P55290	cadherin-13
Q8WTV0	scavenger receptor class B member 1
P27701	CD82 antigen
Q16769	glutaminyl-peptide cyclotransferase
Q9HC38	glyoxalase domain-containing protein
P52565	rho GDP-dissociation inhibitor 1
P14618	pyruvate kinase
P36955	pigment epithelium-derived factor
O00560	syntenin-1
P0DOX3	immunoglobulin delta heavy chain
P07108	acyl-CoA-binding protein
P27169	serum paraoxonase/arylesterase 1
Q14112	nidogen-2
Q9UBI6	guanine nucleotide-binding protein g(i)/g(s)/g(o) subunit gamma-12
A0A075B6K0	immunoglobulin lambda variable 3-16
P01706	immunoglobulin lambda variable 2-11
Q8WW52	protein FAM151A
Q9NQ38	serine protease inhibitor Kazal-type 5
P02647	apolipoprotein A-I
P07988	pulmonary surfactant-associated protein B
P12931	proto-oncogene tyrosine-protein kinase Src
Q9UGT4	sushi domain-containing protein 2
Q9UBP4	dickkopf-related protein 3
P11217	glycogen phosphorylase, muscle form
P04080	cystatin-B
P28072	proteasome subunit beta type-6
Q9BRF8	serine/threonine-protein phosphatase
P56470	galectin-4
Q86XR7	TIR domain-containing adapter molecule 2
Q86UP6	CUB and zona pellucida-like domain-containing protein 1
O75298	reticulon-2

UniProt ID	Description
P14384	carboxypeptidase M
P08238	heat shock protein HSP 90-beta
Q13443	disintegrin and metalloproteinase domain-containing protein 9
P04216	thy-1 membrane glycoprotein
A0A0C4DH34	immunoglobulin heavy variable 4-28
P32926	desmoglein-3
P63000	Ras-related C3 botulinum toxin substrate 1
Q96RF0	sorting nexin-18
P00918	carbonic anhydrase 2
Q08722	leukocyte surface antigen CD47
P05451	lithostathine-1-alpha
P09467	fructose-1,6-bisphosphatase 1
P16278	beta-galactosidase
A0A0C4DH33	immunoglobulin heavy variable 1-24
P29218	inositol monophosphatase 1
P35606	coatomer subunit beta
Q16706	alpha-mannosidase 2
Q16820	meprin A subunit beta
Q14126	desmoglein-2
P84243	histone H3.3
P22304	iduronate 2-sulfatase
P01703	immunoglobulin lambda variable 1-40
O95967	EGF-containing fibulin-like extracellular matrix protein 2
P22079	lactoperoxidase
P32189	glycerol kinase
P06753	tropomyosin alpha-3 chain
O00161	synaptosomal-associated protein 23
P28074	proteasome subunit beta type-5
P16083	ribosyldihydronicotinamide dehydrogenase
Q1EHB4	sodium-coupled monocarboxylate transporter 2
O95274	ly6/PLAUR domain-containing protein
P35556	fibrillin-2
Q6PIF6	unconventional myosin-VIIb
Q02505	mucin-3A
Q5SRE5	nucleoporin NUP188 homolog
P01215	glycoprotein hormones alpha chain
Q15323	keratin, type I cuticular Ha1
P11021	78 kDa glucose-regulated protein
Q12841	follistatin-related protein 1
P02511	alpha-crystallin B chain

© 2019 Heath M et al. *JAMA Network Open.*

eTable 16 *(continued)*. Proteins Identified in Preterm Gut Lumen (N = 635)

Shown are descriptions and Uniprot ID of proteins with MS sumPEP scores of 5.4 to 2.9.

UniProt ID	Description
P02792	ferritin light chain
P59665	neutrophil defensin 1
Q9Y6W3	calpain-7
P35243	recoverin
B0FP48	uroplakin-3b-like protein 1
P63098	calcineurin subunit B type 1
P04075	fructose-bisphosphate aldolase A
A0A075B6H9	immunoglobulin lambda variable 4-69
O75351	vacuolar protein sorting-associated protein 4B
P15924	desmoplakin
P40926	malate dehydrogenase, mitochondrial
Q7Z404	transmembrane channel-like protein
P06681	complement C2
Q9UM44	HERV-H LTR-associating protein 2
A5D6W6	fat storage-inducing transmembrane protein 1
Q96C23	aldose 1-epimerase
P29279	connective tissue growth factor
O95436	sodium-dependent phosphate transport protein 2B
Q8NCW5	NAD(P)H-hydrate epimerase
P35080	profilin-2
O00462	beta-mannosidase
Q16787	laminin subunit alpha-3
P01709	immunoglobulin lambda variable 2-8
P08670	vimentin
P06703	protein S100-A6
O75015	low affinity immunoglobulin gamma Fc region receptor III-B
Q9BXD5	N-acetylneuraminate lyase
P08134	rho-related GTP-binding protein RhoC
P54727	UV excision repair protein RAD23 homolog B
Q5QNW6	histone H2B type 2-F
P27449	V-type proton ATPase 16 kDa proteolipid subunit
O15145	actin-related protein 2/3 complex subunit 3
Q9UKN1	mucin-12
O60235	transmembrane protease serine 11D
O75629	protein CREG1
P99999	cytochrome c
Q8N5I2	arrestin domain-containing protein 1
P19021	peptidyl-glycine alpha-amidating monooxygenase
P80370	protein delta homolog 1
Q16881	thioredoxin reductase 1, cytoplasmic
Q96P63	serpin B12
P05164	myeloperoxidase
O94832	unconventional myosin-I
Q9Y6E0	serine/threonine-protein kinase 24
P50120	retinol-binding protein 2
P23109	AMP deaminase 1
P01033	metalloproteinase inhibitor 1
Q7L5L3	lysophospholipase D GDPD3
Q02487	desmocollin-2
Q6UXV4	MICOS complex subunit MIC27
Q9H1C7	cysteine-rich and transmembrane domain-containing protein 1
Q99816	tumor susceptibility gene 101 protein
O00115	deoxyribonuclease-2-alpha
P13473	lysosome-associated membrane glycoprotein 2
A0A0J9YX35	immunoglobulin heavy variable 3-64D
P61204	ADP-ribosylation factor 3
O96009	napsin-A
P50443	sulfate transporter
P55064	aquaporin-5
A0A0A0MS14	immunoglobulin heavy variable 1-45
Q99436	proteasome subunit beta type-7
P11717	cation-independent mannose-6-phosphate receptor
P48637	glutathione synthetase
Q9H0E2	Toll-interacting protein
P52758	2-iminobutanoate/2-iminopropanoate deaminase
Q9UBC9	small proline-rich protein 3
A0A075B6J9	immunoglobulin lambda variable 2-18
P46459	vesicle-fusing ATPase
P62330	ADP-ribosylation factor 6
A0A075B6I0	immunoglobulin lambda variable 8-61
P17948	vascular endothelial growth factor receptor 1

© 2019 Heath M et al. *JAMA Network Open.*

eFigure 1. Control Experiments Demonstrated Operator Reproducibility, Antibody Reagent Specificity, and Biospecimen Specificity

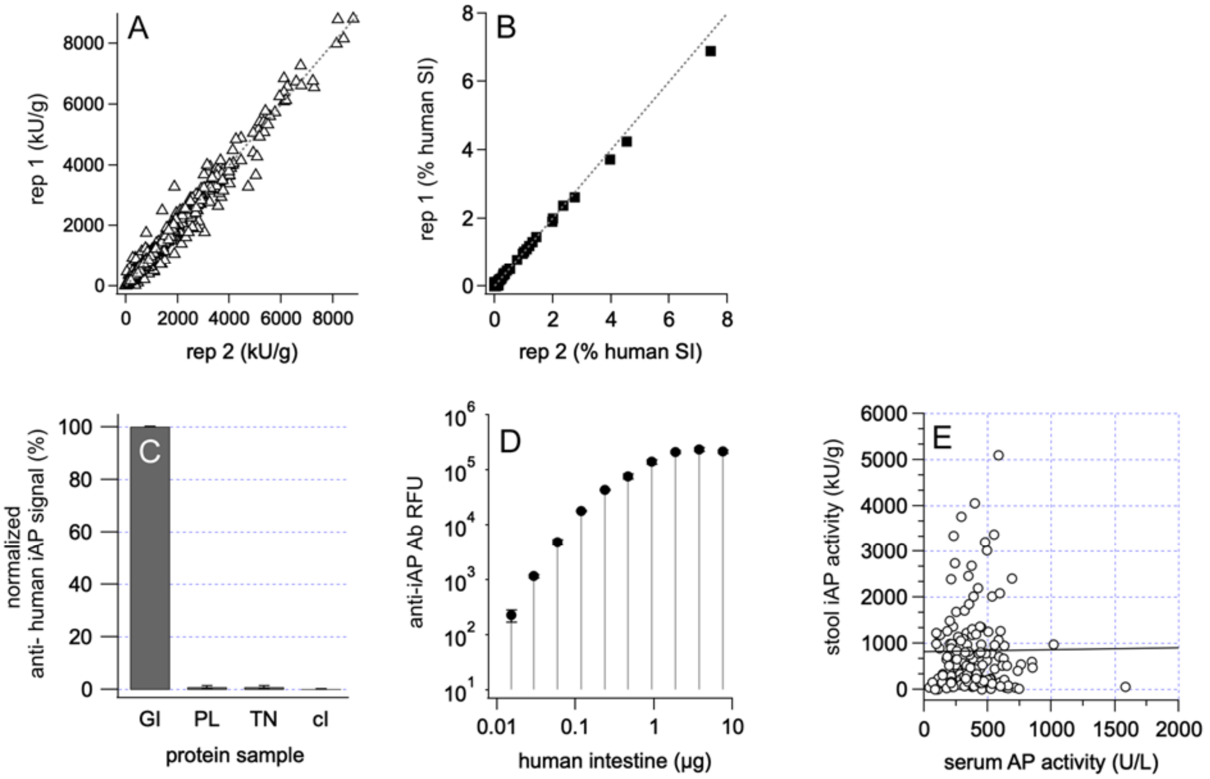

Five different operators performed ***(A)*** activity assay measurements and ***(B)*** iAP content determinations in the patient stool samples; dotted line marks the 1:1 correspondence between replicate 1 and replicate 2. ***(C)*** The anti-human iAP antibody used in this study was tested against human small intestine lysate (GI), purified human placental alkaline phosphatase (PL), purified human tissue non-specific alkaline phosphatase (TN), and bovine intestinal alkaline phosphatase (cI). Quantitation from densitometry for the total amount of AP is listed as the average and SE: 100.0 ± 0.1% (GI); 1.0 ± 0.4% (PL); 0.9 ± 0.5% (TN); 0.3 ± 0.1% (cl); N=5. ***(D)*** Quantitation of immunoblot method used had a linear response to the amount of human intestinal alkaline phosphatase.[5-7] The mean relative fluorescence units and standard error for the total iAP (open triangles) 216,692 ± 14,533 for 7.5 µg; 233,533 ± 20,264 for 3.75 µg; 211,176 ± 132,267 for 1.875 µg; 142,834 ± 13,019 for 0.938 µg; 75,727 ± 7,637 for 0.469 µg; 44,101 ± 1,410 for 0.234 µg; 18,234 ± 450 for 0.117 µg; 4,918 ± 549 for 0.059 µg; 1,164 ± 79 for 0.029 µg; and 227 ± 57 for 0.015 µg. ***(E)*** Comparison of serum AP activity and stool iAP activity, if serum clinical test and stool sample were collected on the sample day. No relationship was observed between serum AP activity and stool iAP activity measurements. N=148; solid line is best linear fit between stool iAP activity and serum AP activity.

© 2019 Heath M et al. *JAMA Network Open.*

eFigure 2. Sequence Alignment of 4 Human Alkaline Phosphatases and Calf Intestinal Alkaline Phosphatase

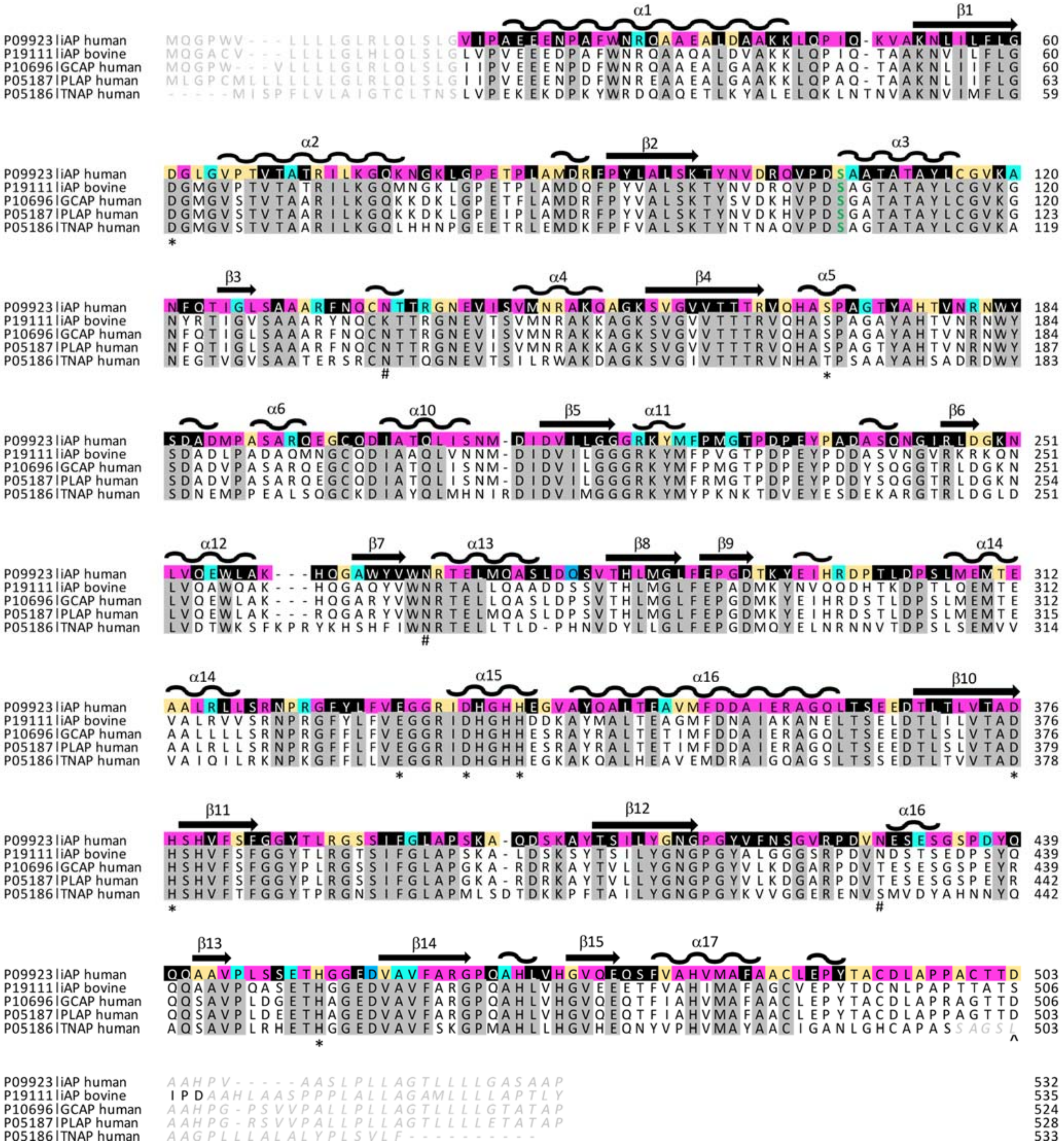

Sequences were shown are human intestinal alkaline phosphatase (iAP human; P09923 Uniprot ID), calf intestinal alkaline phosphatase (iAP bovine; P19111), germ cell alkaline phosphatase (GCAP human; P10696), placenta-like alkaline phosphatase (PLAP human, P05187), and tissue-nonspecific alkaline phosphatase (TNAP human, P05186). Signal peptide is in grey at the N-terminus of the sequences. Propeptide is in grey italics at the C-terminus of the sequences. Residues involved in metal binding are annotated with an asterisk; candidate glycosylation sites have a # symbol. Secondary structure motifs are shown from the human placenta alkaline phosphatase crystals (PDB ID 1EW2). Color heat map of number of polymorphisms found in the human population are overlaid on the IAP human sequence.

© 2019 Heath M et al. *JAMA Network Open.*

eReferences

1. Gupta A, Paria A. Etiology and medical management of NEC. *Early Hum Dev.* 2016;97:17-23.

2. Gephart SM, Gordon PV, Penn AH, et al. Changing the paradigm of defining, detecting, and diagnosing NEC: Perspectives on Bell's stages and biomarkers for NEC. *Semin Pediatr Surg.* 2018;27(1):3-10.

3. Center for Disease Control and Prevention. CDC/NHSN surveillance definitions for specific types of infections. In *2018 NHSN Patient Safety Component Manual*. US Department of Health and Human Services National Healthcare Safety Network, 2018 edition:17.11-17.30.

4. Singer M, Deutschman CS, Seymour CW, et al. The third international consensus definitions for sepsis and septic shock (Sepsis-3). *JAMA.* 2016;315(8):801-810.

5. Henthorn PS, Raducha M, Edwards YH, et al. Nucleotide and amino acid sequences of human intestinal alkaline phosphatase: close homology to placental alkaline phosphatase. *Proc Natl Acad Sci U S A.* 1987;84(5):1234-1238.

6. Henthorn PS, Raducha M, Kadesch T, Weiss MJ, Harris H. Sequence and characterization of the human intestinal alkaline phosphatase gene. *J Biol Chem.* 1988;263(24):12011-12019.

7. Miki K, Suzuki H, Iino S, Oda T, Hirano K, Sugiura M. Human fetal intestinal alkaline phosphatase. *Clin Chim Acta.* 1977;79(1):21-30.

8. Shah J, Singhal N, da Silva O, et al. Intestinal perforation in very preterm neonates: risk factors and outcomes. *J Perinatol.* 2015;35(8):595-600.

9. Attridge JT, Herman AC, Gurka MJ, Griffin MP, McGahren ED, Gordon PV. Discharge outcomes of extremely low birth weight infants with spontaneous intestinal perforations. *J Perinatol.* 2006;26(1):49-54.

10. Hornik CP, Benjamin DK, Becker KC, et al. Use of the complete blood cell count in late-onset neonatal sepsis. *Pediatr Infect Dis J.* 2012;31(8):803-807.

11. Wynn JL. Defining neonatal sepsis. *Curr Opin Pediatr.* 2016;28(2):135-140.

12. Marik PE, Taeb AM. SIRS, qSOFA and new sepsis definition. *J Thorac Dis.* 2017;9(4):943-945.

13. Wynn JL, Polin RA. Progress in the management of neonatal sepsis: the importance of a consensus definition. *Pediatr Res.* 2018;83(1-1):13-15.

14. Buhimschi CS, Bhandari V, Hamar BD, et al. Proteomic profiling of the amniotic fluid to detect inflammation, infection, and neonatal sepsis. *PLoS Med.* 2007;4(1):e18.

15. Buhimschi CS, Buhimschi IA, Abdel-Razeq S, et al. Proteomic biomarkers of intra-amniotic inflammation: relationship with funisitis and early-onset sepsis in the premature neonate. *Pediatr Res.* 2007;61(3):318-324.

16. Corazziari E, Staiano A, Miele E, Greco L, Italian Society of Pediatric Gastroenterology H, Nutrition. Bowel frequency and defecatory patterns in children: a prospective nationwide survey. *Clin Gastroenterol Hepatol.* 2005;3(11):1101-1106.

17. Rose C, Parker A, Jefferson B, Cartmell E. The characterization of feces and urine: a review of the literature to inform advanced treatment technology. *Crit Rev Environ Sci Technol.* 2015;45(17):1827-1879.

18. Consortium U. Reorganizing the protein space at the Universal Protein Resource (UniProt). *Nucleic Acids Res.* 2012;40(Database issue):D71-75.

© 2019 Heath M et al. *JAMA Network Open.*

19. Verberkmoes NC, Russell AL, Shah M, et al. Shotgun metaproteomics of the human distal gut microbiota. *ISME J.* 2009;3(2):179-189.

20. Mahowald MA, Rey FE, Seedorf H, et al. Characterizing a model human gut microbiota composed of members of its two dominant bacterial phyla. *Proc Natl Acad Sci U S A.* 2009;106(14):5859-5864.

21. Erickson AR, Cantarel BL, Lamendella R, et al. Integrated metagenomics/metaproteomics reveals human host-microbiota signatures of Crohn's disease. *PLoS One.* 2012;7(11):e49138.

22. Flynn MA, Gehrke C, Maier BR, Tsutakawa RK, Hentges DJ. Effect of diet on fecal nutrients. *J Am Diet Assoc.* 1977;71(5):521-526.

23. Beyer PL, Flynn MA. Effects of high- and low-fiber diets on human feces. *J Am Diet Assoc.* 1978;72(3):271-277.

24. Cummings JH, Branch W, Jenkins DJ, Southgate DA, Houston H, James WP. Colonic response to dietary fibre from carrot, cabbage, apple, bran. *Lancet.* 1978;1(8054):5-9.

25. Stephen AM, Wiggins HS, Cummings JH. Effect of changing transit time on colonic microbial metabolism in man. *Gut.* 1987;28(5):601-609.

26. Janes KA. An analysis of critical factors for quantitative immunoblotting. *Sci Signal.* 2015;8(371):rs2.

27. Peterson BW, Sharma PK, van der Mei HC, Busscher HJ. Bacterial cell surface damage due to centrifugal compaction. *Appl Environ Microbiol.* 2012;78(1):120-125.

28. Livshits MA, Khomyakova E, Evtushenko EG, et al. Isolation of exosomes by differential centrifugation: Theoretical analysis of a commonly used protocol. *Sci Rep.* 2015;5:17319.

29. Goldberg RF, Austen WG, Jr., Zhang X, et al. Intestinal alkaline phosphatase is a gut mucosal defense factor maintained by enteral nutrition. *Proc Natl Acad Sci U S A.* 2008;105(9):3551-3556.

30. PetitClerc C. Quantitative fractionation of alkaline phosphatase isoenzymes according to their thermostability. *Clin Chem.* 1976;22(1):42-48.

31. O'Brien PJ, Herschlag D. Alkaline phosphatase revisited: hydrolysis of alkyl phosphates. *Biochemistry.* 2002;41(9):3207-3225.

32. Bale JR, Chock PB, Huang CY. The nature of negative cooperativity in alkaline phosphatase: kinetic patterns contrary to the flip-flop model. *J Biol Chem.* 1980;255(18):8424-8430.

33. Fernley HN, Walker PG. Kinetic behaviour of calf-intestinal alkaline phosphatase with 4-methylumbelliferyl phosphate. *Biochem J.* 1965;97(1):95-103.

34. Levine MN, Raines RT. Sensitive fluorogenic substrate for alkaline phosphatase. *Anal Biochem.* 2011;418(2):247-252.

35. Olson BJ, Markwell J. Assays for determination of protein concentration. *Curr Protoc Protein Sci.* 2007;Chapter 3:Unit 3 4.

36. Bradford MM. A rapid and sensitive method for the quantitation of microgram quantities of protein utilizing the principle of protein-dye binding. *Anal Biochem.* 1976;72:248-254.

37. Zor T, Selinger Z. Linearization of the Bradford protein assay increases its sensitivity: theoretical and experimental studies. *Anal Biochem.* 1996;236(2):302-308.

38. Brody JP, Williams BA, Wold BJ, Quake SR. Significance and statistical errors in the analysis of DNA microarray data. *Proc Natl Acad Sci U S A.* 2002;99(20):12975-12978.

© 2019 Heath M et al. *JAMA Network Open.*

39. Tricarico C, Pinzani P, Bianchi S, et al. Quantitative real-time reverse transcription polymerase chain reaction: normalization to rRNA or single housekeeping genes is inappropriate for human tissue biopsies. *Anal Biochem.* 2002;309(2):293-300.

40. Towbin H, Staehelin T, Gordon J. Electrophoretic transfer of proteins from polyacrylamide gels to nitrocellulose sheets: procedure and some applications. *Proc Natl Acad Sci U S A.* 1979;76(9):4350-4354.

41. Burnette WN. "Western blotting": electrophoretic transfer of proteins from sodium dodecyl sulfate--polyacrylamide gels to unmodified nitrocellulose and radiographic detection with antibody and radioiodinated protein A. *Anal Biochem.* 1981;112(2):195-203.

42. Spinola SM, Cannon JG. Different blocking agents cause variation in the immunologic detection of proteins transferred to nitrocellulose membranes. *J Immunol Methods.* 1985;81(1):161-165.

43. Lee JW, Devanarayan V, Barrett YC, et al. Fit-for-purpose method development and validation for successful biomarker measurement. *Pharm Res.* 2006;23(2):312-328.

44. Leek JT, Scharpf RB, Bravo HC, et al. Tackling the widespread and critical impact of batch effects in high-throughput data. *Nat Rev Genet.* 2010;11(10):733-739.

© 2019 Heath M et al. *JAMA Network Open.*
